# Scleraxis is a transcriptional activator that regulates the expression of Tenomodulin, a marker of mature tenocytes and ligamentocytes

**DOI:** 10.1038/s41598-018-21194-3

**Published:** 2018-02-16

**Authors:** Chisa Shukunami, Aki Takimoto, Yuriko Nishizaki, Yuki Yoshimoto, Seima Tanaka, Shigenori Miura, Hitomi Watanabe, Tetsushi Sakuma, Takashi Yamamoto, Gen Kondoh, Yuji Hiraki

**Affiliations:** 1 Department of Molecular Biology and Biochemistry, Division of Dental Sciences, Graduate School of Biomedical and Health Sciences, Hiroshima University, Hiroshima, 734-8553 Japan; 2 Laboratory of Cellular Differentiation, Institute for Frontier Life and Medical Sciences, Kyoto University, Kyoto, 606-8507 Japan; 3 Laboratory of Integrative Biological Science, Institute for Frontier Life and Medical Sciences, Kyoto University, Kyoto, 606-8507 Japan; 4 Department of Mathematical and Life Sciences, Graduate School of Science, Hiroshima University, Higashi-Hiroshima, Hiroshima, Japan; 5 Present Address: Functional Morphology Laboratory, Department of Clinical Pharmacy, Faculty of Pharmacy, Yokohama University of Pharmacy, Yokohama, Japan

## Abstract

Tenomodulin (Tnmd) is a type II transmembrane glycoprotein predominantly expressed in tendons and ligaments. We found that scleraxis (Scx), a member of the Twist-family of basic helix-loop-helix transcription factors, is a transcriptional activator of *Tnmd* expression in tenocytes. During embryonic development, *Scx* expression preceded that of *Tnmd*. *Tnmd* expression was nearly absent in tendons and ligaments of *Scx*-deficient mice generated by transcription activator-like effector nucleases-mediated gene disruption. *Tnmd* mRNA levels were dramatically decreased during serial passages of rat tenocytes. *Scx* silencing by small interfering RNA significantly suppressed endogenous *Tnmd* mRNA levels in tenocytes. Mouse *Tnmd* contains five E-box sites in the ~1-kb 5′-flanking region. A 174-base pair genomic fragment containing a TATA box drives transcription in tenocytes. Enhancer activity was increased in the upstream region (−1030 to −295) of *Tnmd* in tenocytes, but not in NIH3T3 and C3H10T1/2 cells. Preferential binding of both Scx and Twist1 as a heterodimer with E12 or E47 to CAGATG or CATCTG and transactivation of the 5′-flanking region were confirmed by electrophoresis mobility shift and dual luciferase assays, respectively. Scx directly transactivates *Tnmd* via these E-boxes to positively regulate tenocyte differentiation and maturation.

## Introduction

Human and mouse tenomodulin (Tnmd) are type II transmembrane glycoproteins consisting of 317 amino acids^1,2^. Tnmd is a molecule related to chondromodulin (Chmd), which was previously identified from bovine epiphyseal cartilage as a soluble growth/differentiation-promoting factor for rabbit chondrocytes and later as an anti-angiogenic factor^3–5^. The C-terminal cysteine-rich domain containing 120 amino acids is secreted from chondrocytes as mature Chmd after cleavage at the processing signal of the human or mouse Chmd precursor protein containing 334 amino acids. *Tnmd* expression is detected in dense connective tissues, including tendons, ligaments, fascia of skeletal muscle, outer annulus fibrosus of the intervertebral disc, and cornea and sclera of the eye^6–10^, whereas *Chmd* is specifically expressed in avascular hyaline cartilage of the cartilaginous bone primordia during development and growth^3,9,11^ and in articular cartilage^12^. In the postnatal heart, Tnmd and Chmd act as angiogenesis inhibitors and are predominantly localized to the tendinous chord and cardiac valves, respectively^13,14^.

Both Tnmd and Chmd exert anti-angiogenic actions through the homologous C-terminal domain containing eight cysteine residues that form four disulphide bonds^6,7^. In human umbilical vein endothelial cells (HUVECs), proliferation, migration, adhesion to vitronectin, and tube formation on Matrigel were significantly inhibited by adenoviral overexpression of mature Chmd or by overexpression of the C-terminal 116-amino acid fragment of Tnmd containing a secretion signal sequence^6,7^. The growth of solid tumours, such as malignant melanoma in syngeneic mice, was significantly suppressed because of anti-angiogenic activities of mature Chmd or the C-terminal 116-amino acid fragment of Tnmd^6^. Vascular endothelial growth factor-A stimulated lamellipodial extensions and motility of HUVECs was impaired by Chmd^15^. Although loss of *Tnmd* does not cause any apparent anti-angiogenic-related phenotype in mouse embryos^16^, surgical removal of the Tnmd-rich layer from the cardiac tendineae cordis induces angiogenesis and matrix metalloproteinase activation^14^. Double immunostaining of CD31, a cell surface marker of vascular endothelial cells, and Tnmd or Chmd in the mouse forelimb at embryonic day (E) 16.5 revealed that Tnmd and Chmd proteins specifically localize to the avascular region of tendon and cartilage, respectively^9^. Thus, both Tnmd and Chmd are anti-angiogenic molecules specifically expressed in association with avascular mesenchymes.

Tendons and ligaments are categorized into typical dense connective tissues characterised by the regular alignment of thick collagen fibres mainly consisting of type I collagen^17^. Tendons bind skeletal muscles to bones to transmit the mechanical force of contraction, whereas ligaments connect bones to correctly align skeletal elements and reinforce flexible joints. During the embryonic development of musculoskeletal components, *Tnmd* is predominantly expressed in mature tendon fibroblasts (tenocytes) and ligament fibroblasts (ligamentocytes) at high levels^8,18^. Analysis of *Tnmd*-deficient mice revealed that *Tnmd* is necessary for tenocyte proliferation and maturation of tendinous tissue^16^ as well as proliferation and senescence of tendon stem/progenitor cells^19^. In the periodontal ligament that connects the cementum of the tooth and alveolar bone, Tnmd has been demonstrated to enhance the cellular adhesion of fibroblasts^20^. *Tnmd* expression in chick leg tendons is positively regulated by scleraxis (Scx), a basic helix-loop-helix (bHLH) transcription factor that marks both tendon progenitors and tenocytes during chick and mouse embryonic development^8^. In mice lacking *Scx*, Tnmd expression is undetectable in tendons^10,21^; however, it remains unclear whether Scx directly regulates *Tnmd* expression at the transcription level.

In the present study, we investigated how *Scx* regulates *Tnmd* expression under both *in vivo* and *in vitro* conditions. *Tnmd* expression is nearly absent in both the tendons and ligaments of *Scx*-deficient mice generated by transcription activator-like effector nuclease (TALEN)-induced double-strand DNA break. Silencing of *Scx* in tenocytes by small interfering RNA markedly suppressed *Tnmd* mRNA levels. We further analysed the ~1-kb 5′-flanking region and 5′ untranslated region of the mouse *Tnmd* gene that spans 15 kb and is comprised of seven exons. The investigated region contains a TATA promoter and five E-box sites. We identified transactivation of the promoter and its upstream region by Scx and/or E12 or E47 as well as the preferential binding of Scx/E12 or Scx/E47 heterodimers to CAGATG and CATCTG. Similar transactivation and binding to these E-boxes were observed when we tested Twist1/E12 or Twist1/E47 heterodimers. Hence, Scx directly transactivates *Tnmd* via these E-box sites to positively regulate tenocyte/ligamentocyte maturation during development and growth.

## Results

### Expression of *Tnmd* in tenocytes *in vivo* and *in vitro*

**Figure 1:**
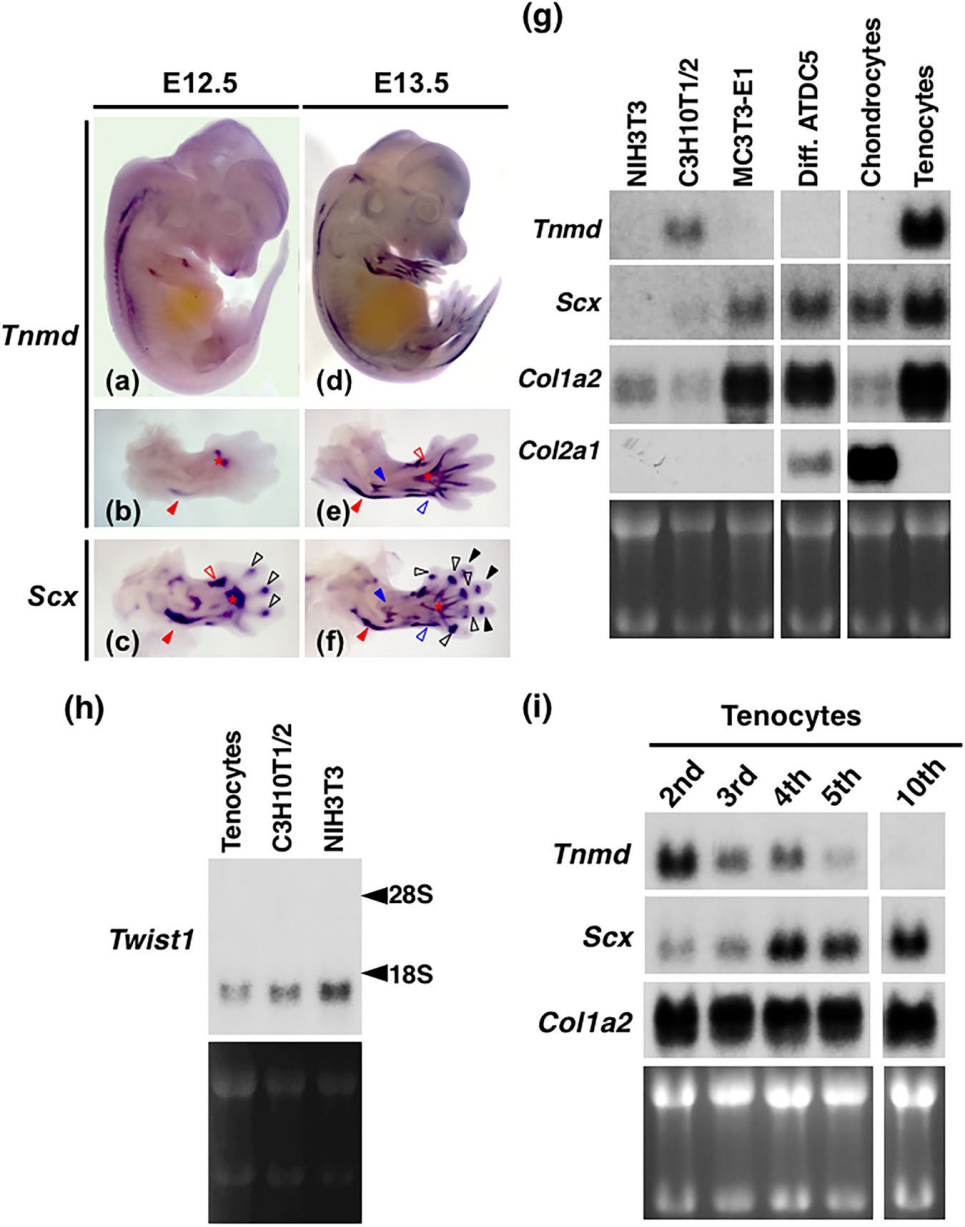
Coexpression of *Tnmd* and *Scx* in the developing tendons. (**a**–**f**) Expression patterns of *Tnmd* (**a**,**b**,**d**,**e**) and *Scx* (**c**,**f**) in embryos at E12.5 (**a**–**c**) and E13.5 (**d**–**f**) are shown. Whole-mount *in situ* hybridisation with antisense probes for these two genes were performed. Lateral (**a**,**d**) and dorsal views of the forelimbs (**b**,**c**,**e**,**f**) are shown, respectively. Red, black, and blue arrowheads indicate the developing triceps branchii tendon, joint capsules between proximal and middle phalanx, and extensor digitorum communis, respectively. Open red, black, and blue arrowheads indicate the developing extensor carpi radialis brevis/longus tendon, joint capsules between the metacarpus and proximal phalanx, and extensor carpi radialis tendon, respectively. Asterisks (**b**,**c**,**e**,**f**) indicate the extensor digitorum communis tendon. (**g**) Total RNA was prepared from NIH3T3 cells on day 3, C3H10T1/2 cells on day 3, MC3T3-E1 cells on day 14, differentiated ATDC5 cells on day 21 (Diff. ATDC5), primary rat costal chondrocytes on day 24 (Chondrocytes), and a secondary culture of rat limb tendon-derived tenocytes (Tenocytes). Detection of *Tnmd*, *Scx*, *Col1a2*, or and *Col2a1* by northern blotting is shown. (**h**) Detection of *Twist1* by northern blotting is shown. Arrowheads indicate the positions of ribosomal RNA subunits. (**i**) Total RNA was extracted from confluent 2nd, 3rd, 4th, 5th, or 10th passages of tenocytes isolated from rat leg tendons. Northern blot analysis of *Tnmd*, *Scx*, and *Col1a2* is shown. Total RNA (15 µg) was loaded in each lane and the loading levels were verified by ethidium bromide staining.

*Tnmd* is predominantly expressed in dense connective tissue, such as tendons and ligaments^1,2,8,9,18^. We compared the expression of *Scx* and *Tnmd* in the developing mouse embryos at E12.5 and E13.5 by whole-mount *in situ* hybridisation (Fig. 1a–f). At E12.5, *Tnmd* was detected in the developing axial tendons along the cervical and thoracic spine, but its expression was low in the limbs (Fig. 1a). In the developing forelimb, *Tnmd* was detected in the primordia of the triceps brachii tendon and extensor digitorum communis tendon (Fig. 1b), while *Scx* was widely detected in the tendon primordia of the forelimb (Fig. 1c). At E13.5, *Tnmd* was expressed in the axial tendons along the entire spine and limbs (Fig. 1d). *Tnmd* and *Scx* were coexpressed with the developing tendons (Fig. 1e,f); however, *Scx* expression was also detected in the developing joint capsules at high levels (Fig. 1f).

We also analysed the expression of *Tnmd* along with other differentiation markers, including *Scx*, *collagen alpha-2*(*I*) *chain* (*Col1a2*), and *collagen alpha-1*(*II*) *chain* (*Col2a1*), in various mesenchymal cells (Fig. 1g, Supplementary Fig. 1). *Tnmd* mRNA was detected at high levels in tenocytes and to a lesser extent in C3H10T1/2 cells, whereas *Tnmd* expression was undetectable in fibroblastic NIH3T3 cells, osteoblastic MC3T3-E1 cells, chondrogenic ATDC5 cells, or primary costal chondrocytes by northern blot analysis (Fig. 1g). In contrast, *Scx*, a marker of both tendon progenitors and tenocytes^22^, was expressed in tenocytes, C3H10T1/2 cells, MC3T3-E1 cells, differentiated ATDC5 cells, and chondrocytes but not in NIH3T3 cells, suggesting that not only tendon-derived cells but also skeletal cells express *Scx in vitro* (Fig. 1g). We also detected the expression of *Twist1* in rat tenocytes, NIH3T3 cells, and C3H10T1/2 cells (Fig. 1h, Supplementary Fig. 2). Interestingly, the mRNA level of *Tnmd* dramatically decreased during serial passaging of rat tenocytes, while increased mRNA levels of *Scx* were observed in the fourth or later culture passages of tenocytes and high levels of *Col1a2* were detected during serial passages until the 10^th^ culture (Fig. 1i, Supplementary Fig. 3). These results clearly suggest that *Tnmd* is a specific marker gene for tenocytes both *in vivo* and *in vitro*.

### Indispensable role of *Scx* in expression of *Tnmd* gene and protein in tendons and ligaments

**Figure 2:**
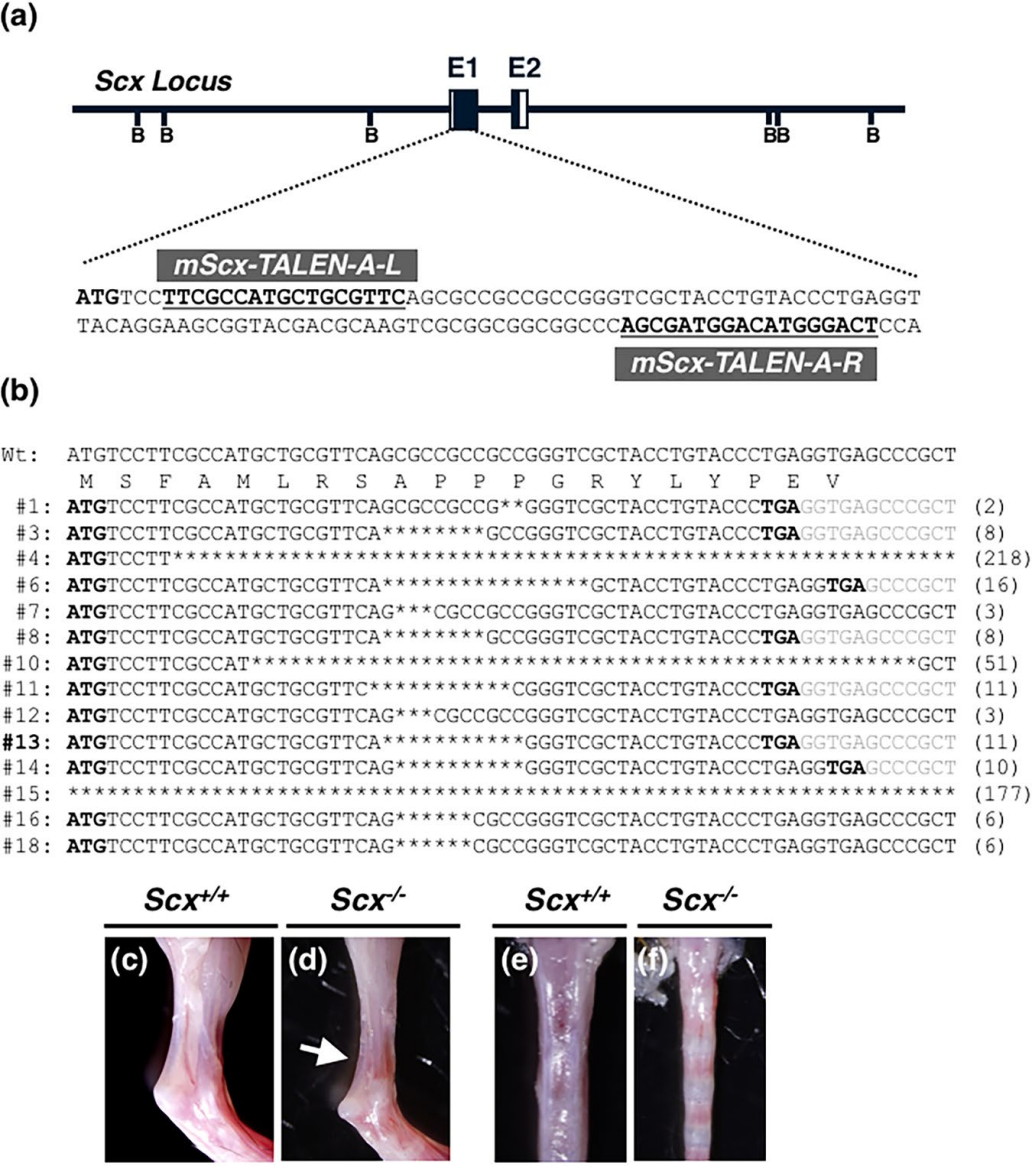
Generation of mice lacking *Scx* by TALENs. (**a**) Genomic structure of *Scx* and TALEN target sequences in the mouse *Scx* locus. Sequences immediately downstream of the initiation codon are shown. The initiation codon of *Scx* is shown in bold text. The left (L) and right (R) binding regions of *mScx-TALEN-A* are indicated by bold and underlined text. BamHI (B) sites are shown on the map. (**b**) Sequences of wild-type (Wt) and founder mice generated by microinjection of *mScx-TALEN-A-R*/*L* mRNAs. Nucleotide deletions are indicated by asterisks. Generation of both an initiation codon and stop codon resulting from a frameshift mutation is shown in bold text. The numbers of the deleted bases are shown in parentheses. A mouse line obtained from founder #13 was used for the analysis. (**c**–**f**) Achilles and tail tendons of *Scx*^+/+^ (**c**,**e**) and *Scx*^−/−^ (**d**,**f**) mice at 2-weeks of age are shown. The arrow indicates hypoplastic formation of the Achilles tendon in a *Scx*^−/−^ (**d**) mouse.

Mouse *Scx* consists of two exons (Fig. 2a). To generate *Scx*-deficient mice, we used TALEN-mediated technology rather than a conventional homologous recombination-based strategy using embryonic stem cells. To disrupt the *Scx* gene using TALENs, we designed the TALEN recognition sequences to be within exon 1 of the mouse *Scx* locus (Fig. 2a), so that most of the Scx protein would be lost due to a frameshift mutation after creation of a double-stranded break by TALENs. TALEN mRNAs produced by *in vitro* transcription were microinjected into the cytoplasm of fertilized oocytes. After *in vitro* culture of the injected oocytes overnight, two-cell embryos were transferred into pseudopregnant female mice. Fourteen pups with deletion mutations were identified from among 18 newborn mice by genotyping and direct sequencing of the amplified DNA (Fig. 2b). We obtained eight founder mice with deletion mutations that would yield a disruptive frameshift mutation and a premature stop codon (#1, #3, #4, #6, #8, #11, #13, and #14), thereby inactivating the Scx protein (Fig. 2b). We crossed founder #13 with a 11-base pair (bp) deletion, both resulting in frameshift and premature stop codons shortly downstream, with a C57BL/6 wild-type mouse to obtain the heterozygote through germline transmission. Heterozygous *Scx*^+/−^ and homozygous *Scx*^−/−^ mice were viable at two weeks, while *Scx*^−/−^ mice exhibited hypoplastic tendon formation (Fig. 2d,f) compared to wild-type mice (Fig. 2c,e), as previously reported^10,21^.

**Figure 3:**
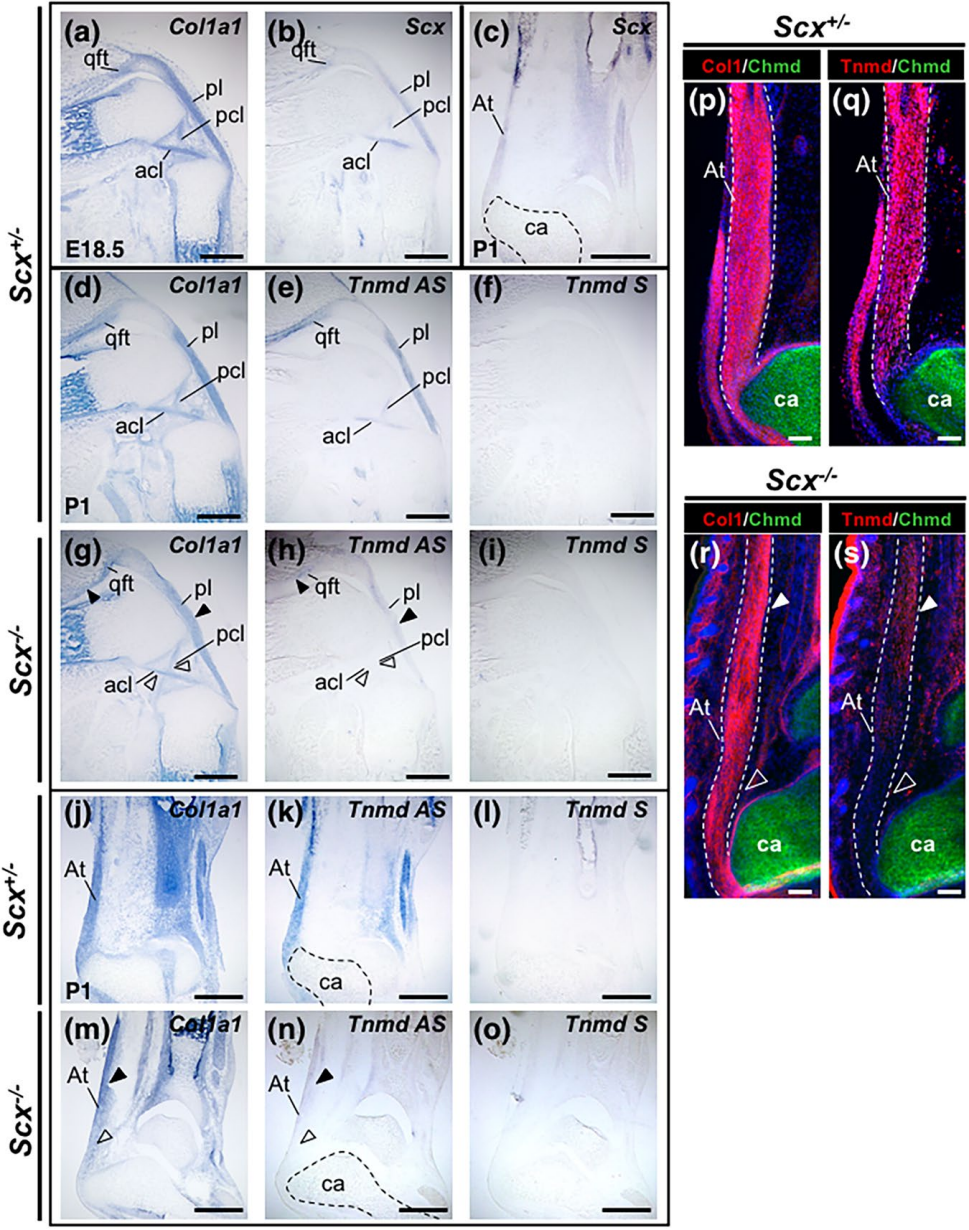
Decreased gene and protein expression of Tnmd in *Scx*-deficient mice. (**a**–**o**) *In situ* hybridisation with antisense probes for *Col1a1* (**a**,**d**,**g**,**j**,**m**), *Scx* (**b**,**c**), and *Tnmd* (**e**,**h**,**k**,**n**) or a sense probe for *Tnmd* (**f**,**i**,**l**,**o**) was performed on semiserial sections prepared from the legs of a *Scx*^+/−^ embryo at E18.5 (**a**,**b**), a *Scx*^+/−^ neonate at P1 (**c**–**f**,**j**–**l**) or a *Scx*^−/−^ neonate at P1 (**g–i**,**m–o**). Sagittal sections of the knee joint (**a**,**b**,**d**–**i**) and the ankle (**c**,**j–o**) are shown. Black and hollowed arrowheads in (**g**,**h**,**m**, and **n**) indicate the tendons/ligaments that were positive or negative staining with the *Tnmd* antisense probe, respectively. (**p–s**) Double immunostaining of Chmd (green) and Col1 (red) (**p**,**r**) or Tnmd (red) (**q**,**s**) was performed on frozen sections prepared from *Scx*^+/−^ (**p**,**q**) and *Scx*^−/−^ (**r**,**s**) at P1. Sagittal sections of the ankle are shown. White and hollowed arrowheads in (**r**,**s**) indicate the regions of Achilles tendon that were positive and negative for staining with the Tnmd antibody, respectively. acl, anterior cruciate ligament; At, Achilles tendon; ca, calcaneus; fe, femur; pa, patella; pcl, posterior cruciate ligament; pl, patella ligament; qft, quadriceps femoris tendon. Scale bars, 500 μm.

To examine the expression of *Tnmd* in *Scx*^+/−^ and *Scx*^−/−^ mouse tissues, we performed *in situ* hybridisation in frozen tissue sections (Fig. 3a–o). In the hindlimb of a *Scx*^+/−^ embryo at E18.5 and a *Scx*^+/−^ neonate at postnatal day 1 (P1), expression of both *Col1a1* (Fig. 3a,j) and *Scx* (Fig. 3b,c) was detected in the tendons and ligaments of the knee (Fig. 3a,b) and ankle (Fig. 3c,j). Similar to *Scx*, *Tnmd* was expressed in *Col1a1-*positive tendons and ligaments of the knee (Fig. 3d,e) and ankle (Fig. 3j,k) of the *Scx*^+/−^ neonate at P1. In the *Scx*^−/−^ neonate at P1, *Col1a1* expression persisted in the tendons and ligaments (Fig. 3g,m), whereas *Tnmd* expression was nearly absent (Fig. 3h,n). Expression of *Tnmd* was undetectable in the anterior and posterior cruciate ligaments and distal region of the Achilles tendon of *Scx*^−/−^ at P1 (Fig. 3h,n). However, only faint expression of *Tnmd* was detected in the quadriceps femoris tendon, patella ligament, and proximal portion of the Achilles tendon of *Scx*^−/−^ at P1 (Fig. 3h,n). We performed immunostaining to examine the localization of Tnmd proteins in the Achilles tendon (Fig. 3p–s). In the ankle of *Scx*^+/−^ and *Scx*^−/−^ at P1, hyaline cartilage of calcaneus was positively stained with an anti-Chmd antibody (Fig. 3p–s) and localization of Col1 was observed in the Achilles tendon (Fig. 3p,r). In good agreement with the expression pattern of mRNA, the Tnmd protein was localized to the Achilles tendon of *Scx*^+/−^ at P1 (Fig. 3q), whereas in the *Scx*^−/−^ neonate, faint or no expression of Tnmd was detected in the proximal or distal portion of the Achilles tendon, respectively (Fig. 3s).

**Figure 4:**
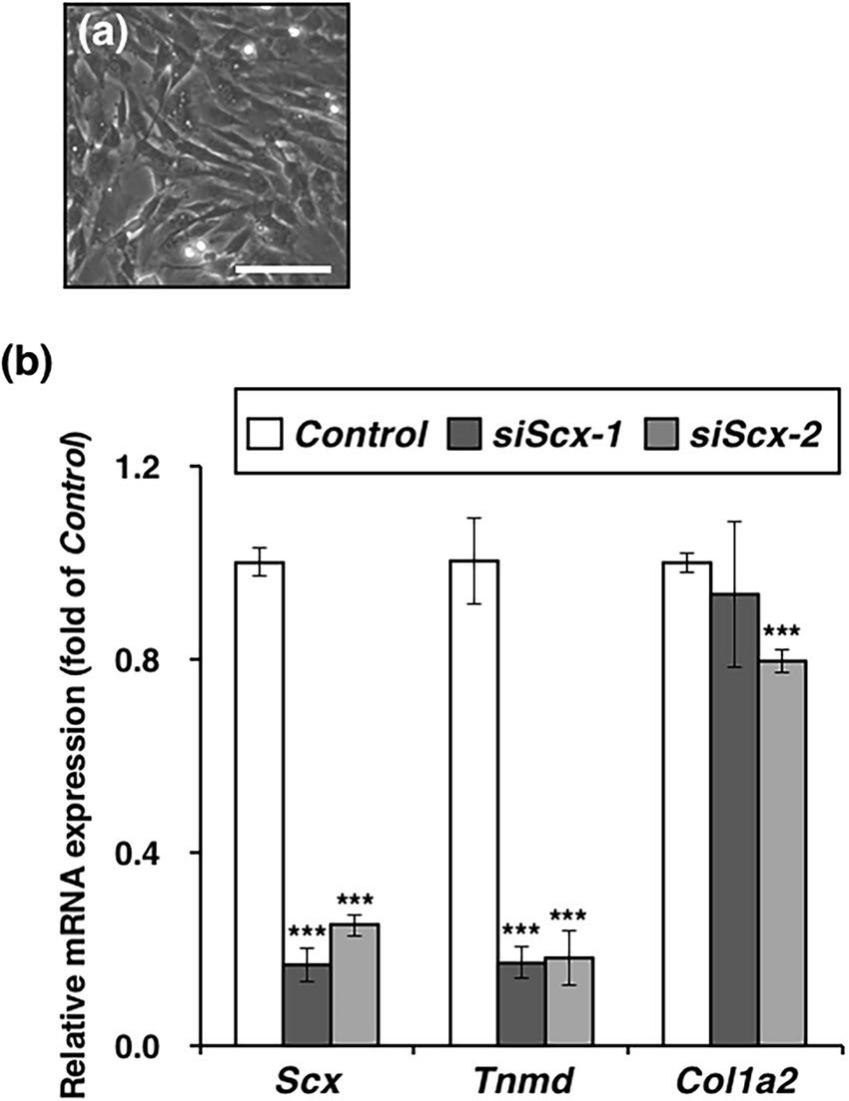
Downregulation of *Tnmd* by knockdown of *Scx* in tenocytes. (**a**) Rat tenocytes were seeded at a density of 3 × 10^4^ cells/well in a 24-well plate and cultured to confluence. (**b**) At 24 h after inoculation, the cells were transfected with non-targeting siRNA (*Control*) or *Scx* siRNAs (*siScx-1* or *siScx-2*) by lipofection. Total RNA was extracted from the cells at 72 h after lipofection and the expression levels of *Scx*, *Tnmd*, or *Col1a2* were examined by qRT-PCR. The data represent the average of 3 independent experiments. The relative expression of each gene is normalized to the control and reported as the mean ± SD. ***P < 0.001 vs. control.

We isolated tenocytes from rat limb tendons (Fig. 4a) and depleted *Scx* in these cells by RNA interference. In tenocytes transfected with *siScx-1* or *siScx-2*, the level of *Scx* was decreased to less than 25% of that of the control at 72 h after lipofection (Fig. 4b). The level of *Tnmd* was markedly decreased to 17% and 18% by gene silencing of *Scx* with *siScx-1* and *siScx-2*, respectively (Fig. 4b). The expression level of *Col1a2* was slightly decreased to 80% by *siScx-2*, but no significant decrease in *Col1a2* expression was detected in cells transfected with *siScx-1* (Fig. 4b). These results suggest that *Tnmd* expression depends on *Scx* in postnatal mature tenocytes.

### Determination of transcription start sites in mouse *Tnmd* gene

**Figure 5:**
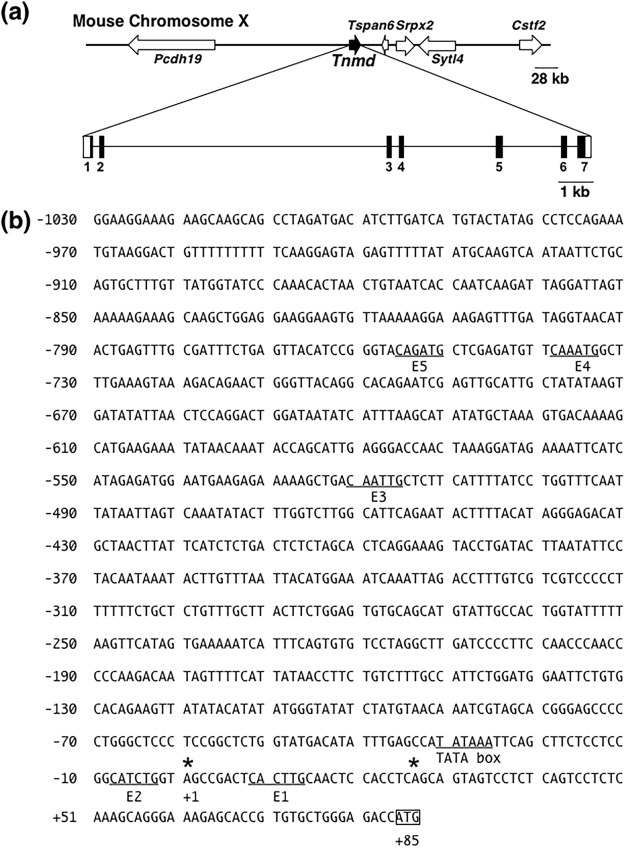
Structure of gene and 5′-flanking region of mouse *Tnmd*. (**a**) Organization of mouse *Tnmd*. Closed and open boxes in the horizontal line represent the coding and untranslated regions of mouse *Tnmd*, respectively. The scale bar represents 1 kilobase (kb). (**b**) Nucleotide sequences of the 5′-flanking region, 5′-untranslated region (5-UTR), and translation start site of mouse *Tnmd*. Asterisks indicate the transcription start sites as determined by CapSite hunting. The translation initiation codon ATG is boxed. A TATA box and the consensus sequences of the E-box (CANNTG) for bHLH transcription factors (E1, E2, E3, E4, and E5) are underlined.

*Tnmd* was cloned as a related gene of Chmd, which was identified as a cartilage-derived angiogenesis inhibitor^1,2^. Mouse *Tnmd* was mapped to chromosome XqE3 (nucleotides 133,851,207–133,865,578 in the UCSC Genome Browser [GRCm38/mm10 assembly]). Based on the genomic and cDNA sequences (GenBank AF219993) of mouse *Tnmd*^2^, we determined the exon-intron boundaries (Table 1) and found that *Tnmd* consists of seven exons spanning approximately 15 kb (Fig. 5a). *Protocadherin* (*Pcdh19*) is located more than 160-kb upstream from the first exon of *Tnmd*. *Tetraspanin 6* (*Tspan6*) is located immediately downstream of *Tnmd*, followed by *sushi repeat-containing protein*, *X-linked 2* (*Srpx2*) and *synaptotagmin-like 4* (*Styl4*) (Fig. 5a).

**Table 1:** Nucleotide sequence at the exon-intron boundaries of mouse *Tnmd*.

Exon 1	CTAAATgtaagt—intron 1—gcttagGCAGAA	Exon 2
Exon 2	AAGAAAgtaagt—intron 2—ttctagGATGTC	Exon 3
Exon 3	AAAAATgtgagt—intron 3—ttatagGGATAC	Exon 4
Exon 4	GATGAGgtatgt—intron 4—tttcagAATGAA	Exon 5
Exon 5	TAGCAGgtatgg—intron 5—tcttagTTTCAG	Exon 6
Exon 6	GACTATgtgagt—intron 6—ttctagACTGAA	Exon 7

Putative splicing donor and acceptor sequences are underlined. Nucleotide sequences of exons and introns are shown in uppercase and lowercase characters, respectively.

As an initial step in the identification and characterisation of the mouse *Tnmd* promoter, we analysed the transcription start site using CapSite hunting technology. We performed nested PCR using CapSite cDNA derived from mouse embryos at E16 and examined six independent cDNA clones. Subsequent sequence analysis revealed two different transcription start sites located at −58 and −84 bp from the translational start site of mouse *Tnmd* (Fig. 5b). Designating the distal transcriptional start site as +1, a putative TATA box was found −25 bp upstream of the distal transcription start site (Fig. 5b). We also found that half of the cDNA clones amplified from CapSite cDNA contained a 90-nucleotide insertion between the cDNA sequences corresponding to exon 2 and exon 3 (data not shown). The inserted region encoding a 30-amino acid sequence was determined to be a single independent exon, located 348-bp upstream of exon 3 in mouse *Tnmd*. The boundary sequences of this inserted region of the genome follow the GT-AG rule. To date, *Tnmd* transcripts containing the insertion sequence have only been found in cDNA clones amplified from mouse 16-day embryo CapSite cDNA.

*Tnmd* expression in tenocytes is positively regulated by Scx, as previously reported^8^. Dimers of various bHLH proteins bind to a short DNA motif known as E-box CANNTG^23^. Five consensus E-box sites are found upstream and downstream of the TATA box of mouse *Tnmd* (Fig. 5b). We designated these E-boxes as E1 (CACTTG), E2 (CATCTG), E3 (CAATTG), E4 (CAAATG), and E5 (CAGATG).

### Promoter activity of the mouse *Tnmd* gene in *Tnmd-*expressing tenocytes

**Figure 6:**
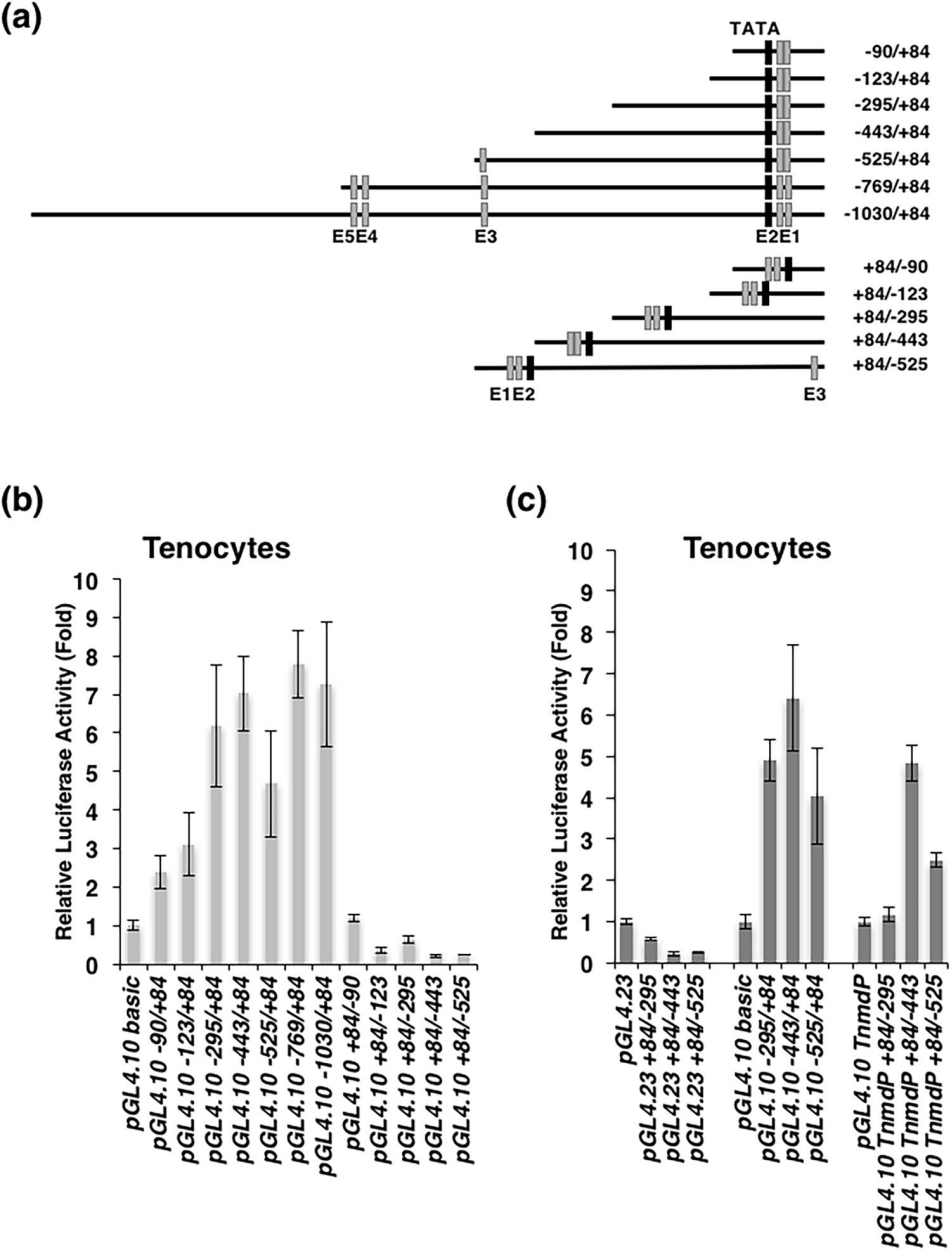
Promoter activity of mouse *Tnmd* in rat tenocytes. (**a**) Various lengths of DNA fragments of mouse *Tnmd* (175–1114 bp) in the forward or reverse orientation were cloned into the promoterless *pGL4*.*10[luc2]*(*pGL4.10 basic*) Vector or *pGL4*.*23[luc2*/*minP](pGL4.23*) with a minimal promoter. The location of the first nucleotide of the upstream transcription start site is denoted as +1. The putative E-box sites and TATA box are shown by grey and black boxes, respectively. (**b**,**c**) Promoter activities of the mouse *Tnmd* gene in *Tnmd*-expressing tenocytes. Cells were co-transfected with a series of constructs shown in (**a**) and *pGL4*.*74[hRluc*/*TK]*. Firefly and Renilla luciferase activities were measured 24 h after transfection. The relative luciferase activity normalized to the *Renilla* luciferase activity is depicted as the fold-induction compared to each activity of the empty vector. All dual luciferase assays were performed in triplicate. The graphs show one representative experiment out of three. Each bar represents the average of three independent transfections (means ± SD.).

**Figure 7:**
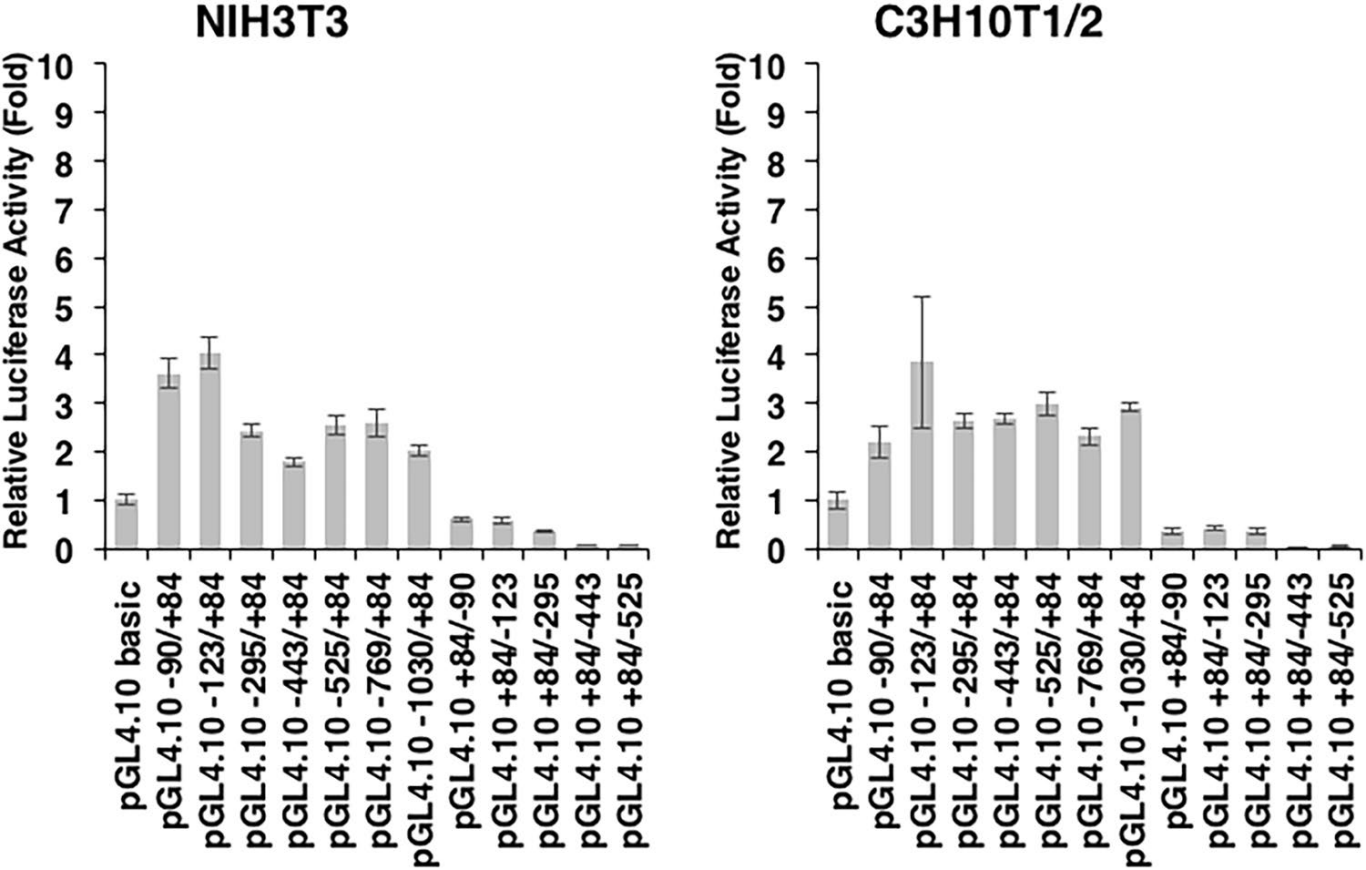
Promoter activity of mouse *Tnmd* in NIH3T3 and C3H10T1/2 cells. NIH3T3 cells and C3H10T1/2 cells on day 2 were co-transfected with a series of constructs and *pGL4*.*74[hRluc*/*TK]*. Firefly and Renilla luciferase activities were measured 24 h after transfection. Relative luciferase activity is depicted as the fold-induction normalized to *Renilla* luciferase activity. All dual luciferase assays were performed in triplicate. The graphs show a representative experiment out of three. Each bar represents the average of three independent transfections (means ± S.D.).

Sequence analysis of the 5′-flanking region revealed a stretch of 746 bp of repetitive sequences (−1762 bp to −1018) containing GT-rich repeats, GA- or CA-rich repeats, and AG- and GG-rich repeats. Thus, we analysed the 5′-flanking region from −1030 to +84 bp containing the TATA box, transcriptional start sites, and five E-boxes (Fig. 5b). To analyse the promoter activity, various lengths of the genomic fragments were cloned into a promoterless *pGL4*.*10[luc2]* vector in the forward or reverse orientation (Fig. 6a). The promoter activity of mouse *Tnmd* was tested by transiently transfecting these constructs into *Tnmd*-expressing tenocytes (Fig. 1g, Supplementary Fig. 1). The luciferase activities of −90/+84 and −123/+84 in tenocytes were 2.4-fold higher than that of the *pGL4*.*10[luc2]* empty vector (Fig. 6b). Increased luciferase activities of −295/+84 (6.2-fold), −443/+84 (7-fold), −525/+84 (4.7-fold), −769/+84 (7.8-fold), and −1030/+84 (7.3-fold) were observed in tenocytes (Fig. 6b). The luciferase activities of +84/−90, +84/−123, +84/−295, +84/−443 and +84/−525 which contained the reverse-oriented genomic fragments were similar or even lower in all tested cells compared to that of the *pGL4*.*10[luc2]* empty vector (Fig. 6b), suggesting that this promoter is active in the forward direction but not in the reverse direction. We also performed dual luciferase assays in *Tnmd*-non-expressing NIH3T3 cells and *Tnmd*-weakly-expressing C3H10T1/2 cells (Figs 1g and 7). A 174-bp promoter region with a TATA box drove the transcription at a similar basal level in tenocytes, NIH3T3 cells, and C3H10T1/2 cells. However, increased enhancer activity in the upstream region (−1030 to −295) was not observed in these cells. Interestingly, as shown in Fig. 6c, the luc activities of −295/+84, −443/+84, and −525/+84 cloned into the promoter-less *pGL4*.*10[luc2]* vector were 4.9-, 6.4-, and 4.0-fold higher than that of the empty vector in tenocytes, whereas the luciferase activities of genomic fragments (+84/−295, +84/−443, and +84/−525) cloned into *pGL4*.*23[luc2*/*minP]* vector with a minimal promoter (AGACACTAGAGGG**TATATAA**TGGAAGCTCGACTTCCAG) were significantly lower than that of the empty vector. When these genomic fragments were cloned into *pGL4*.*10[luc2*/*TnmdP]* with a −90/+84F-containing TATA box, increased luciferase activities were detected in tenocytes harboring −443/+84 (4.8-fold) and −525/+84 (2.5-fold) (Fig. 6c). These results suggest that the TATA box-containing region from −90 to +84 is sufficient for core promoter activity and increased enhancer activity of the upstream region was observed in *Tnmd*-expressing tenocytes, depending on the endogenous *Tnmd* promoter.

### Transactivation of mouse *Tnmd* promoter by Scx or Twist1, which dimerizes with E12 or E47

**Figure 8:**
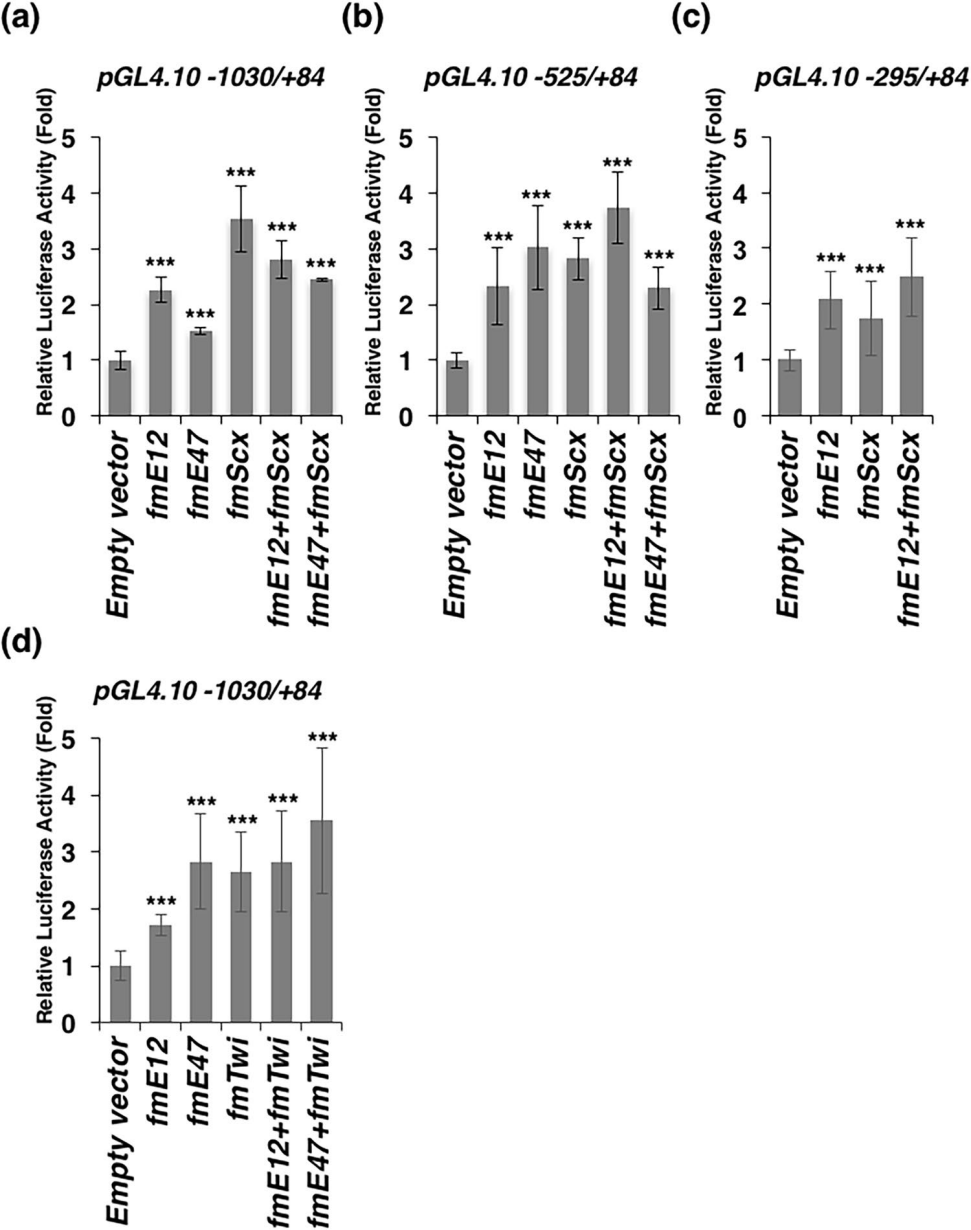
Transactivation of the mouse *Tnmd* 5′-flanking region containing E-boxes by bHLH factors in rat tenocytes. The mouse *Tnmd* genomic fragment containing the 5′-flanking region and 5′-UTR with five E-boxes (from −1030 to +84), three E-box sites (from −525 to +84), or two E-box sites (from −295 to +84) was subcloned into a promoterless *pGL4*.*10[luc2]* in the forward orientation (*pGL4.10 −525/*+*84* or *pGL4.10 −1030/*+*84*). Rat tenocytes were co-transfected with the reporter construct and empty vector *pcDNA3* as a control, or expression vectors encoding the FLAG fusion of mouse E12 (*fmE12*), mouse E47 (*fmE47*), mouse Scx (*fmScx*), mouse Twist1 (*fmTwi*) and *pGL4*.*74[hRluc*/*TK]*. Firefly and Renilla luciferase activities were measured 24 h after transfection. Values were normalized using a *pGL4*.*74[hRluc*/*TK*] and are presented as the fold-induction relative to *pcDNA3* (empty vector). Graphs show one representative experiment of at least three. Each bar represents the average of three independent transfections (means ± SD). ***P < 0.05 vs. control.

Scx is a member of the Twist family of bHLH transcription factors, which functions through dimerization with E-proteins and binding to E-boxes (CANNTG)^24^. Both *Scx* and *Twist1* were co-expressed with *Tnmd* in tenocytes (Fig. 1g,h) and upregulated the expression of *Tnmd* in chick tenocytes, whereas overexpression of *Myog*, a myogenic bHLH factor, resulted in downregulation of the mRNA levels of *Tnmd* in chick tenocytes^25^. To examine whether Scx is directly involved in the transactivation of mouse *Tnmd*, we performed dual luciferase assays in rat tenocytes by co-transfecting the luciferase reporter containing five E-boxes (*pGL4.10 −1030/*+*84*), three E-boxes (*pGL4.10 −525/*+*84*), or two E-boxes (*pGL4.10 −295/*+*84*) with various combinations of expression vectors for FLAG-tagged mouse Scx (fmScx) or Twist1 (fmTwi) and/or its heterodimeric partners E12 (fmE12) or E47 (fmE47) tagged with FLAG (Fig. 8). E12 and E47 are products of two alternatively spliced mRNAs and have nearly identical sequences, except in the stretches encoding the DNA-binding domain^26^. The luciferase activities of *pGL4.10 −1030/*+*84*, *pGL4.10 −525/*+*84*, or *pGL4.10 −295/*+*84* cotransfected with fmScx and/or fmE12 or fmE47 were significantly higher than that in the control (Fig. 8a,b,c). Similar luciferase activity of *pGL4.10 −1030/*+*84* was observed when cotransfected with *fmTwi* and/or *fmE12* or *fmE47* (Fig. 8d).

**Figure 9:**
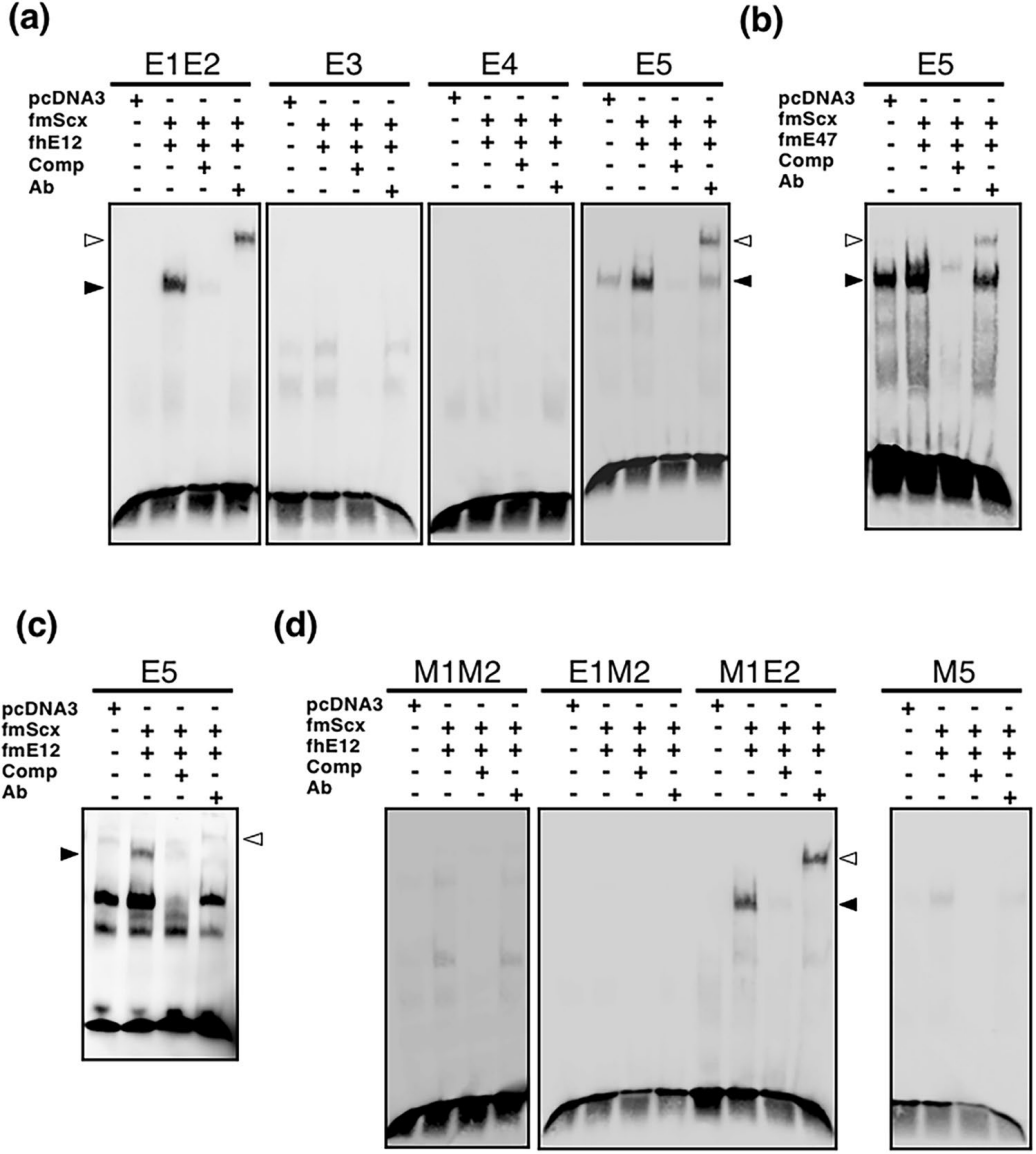
Preferential binding of Scx/E12 or Scx/E47 to E2 and E5. Double-stranded synthetic oligonucleotides containing the E-box were end-labelled with biotin and used as a probe for EMSA. The oligonucleotides sequences used in EMSA (E1E2, M1M2, E1M2, M1E2, E3, E4, E5, and M5) are shown in Table 2. Results shown in (**a**), (**b**), (**c**) and (**d**) are from assays using oligonucleotides containing the consensus E-box sequences (E1E2, E3, E4, and E5) and mutated oligonucleotides (M1M2, E1M2, M1E2, and M5), respectively. Biotin-labelled probes were incubated with nuclear extracts from HEK293T cells transfected with the empty vector *pcDNA3* as a control, or expression vectors encoding the FLAG fusion of human E12 (fhE12), mouse E12 (fmE12), mouse E47 (fmE47), and mouse Scx (fmScx). The binding reactions were performed in the presence or absence of non-labelled oligonucleotides as binding competitors (Comp) or an anti-FLAG antibody (Ab). The positions of shifted and super-shifted bands are shown with black and hollowed arrowheads, respectively.

We then tested whether Scx and E12 or E47 directly interact with these E-boxes identified in the promoter region using electrophoretic mobility shift assays (EMSA). EMSA was performed with nuclear extracts containing fmScx and FLAG-tagged human E12 (fhE12), fmE12, or fmE47 using biotin-labelled oligonucleotides containing the E-box and/or mutated E-box (Table 2). The expected molecular weight of each translated protein was confirmed by western blotting (data not shown). Of the five consensus E-box sequences, a specific shift band was detected when fmScx and fhE12, fmE12 or fmE47 was incubated with a biotin-labelled E1E2 or E5 oligonucleotide (black arrowheads in Fig. 9a–c). In the presence of an anti-FLAG antibody, a supershifted band was clearly detected (hollowed arrowheads in Fig. 9a–c), suggesting that Scx and E12 or E47 heterodimer complexes directly interact with the oligonucleotide E1E2 or E5. To identify which E-box on E1E2 is responsible for the specific binding of Scx/E12, we performed EMSA with mutated oligonucleotides (Table 2, Fig. 9d). Both a specific shift band and supershifted band were detected when fmScx and fhE12 were incubated with the biotin-labelled M1E2 oligonucleotide containing a mutated E1 sequence and normal E2 sequence. No specific bands were detected with the biotin-labelled M1M2, E1M2, or M5 oligonucleotides (Fig. 9d).

**Table 2:** Oligonucleotides for electrophoresis mobility shift assay.

	Sequence
E1E2	5′ CCTCCGG**CATCTG**GTAGCCGACT**CACTTG**CAACT 3′
	3′ GGAGGCC**GTAGAC**CATCGGCTGA**GTGAAC**GTTGA 5′
M1M2	5′ CCTCCGG**TTTTTT**GTAGCCGACT**TTTTTT**CAACT 3′
	3′ GGAGGCC**AAAAAA**CATCGGCTGA**AAAAAA**GTTGA 5′
E1M2	5′ CCTCCGG**TTTTTT**GTAGCCGACT**CACTTG**CAACT 3′
	3′ GGAGGCC**AAAAAA**CATCGGCTGA**GTGAAC**GTTGA 5′
M1E2	5′ CCTCCGG**CATCTG**GTAGCCGACT**TTTTTT**CAACT 3′
	3′ GGAGGCC**GTAGAC**CATCGGCTGA**AAAAAA**GTTGA 5′
E3	5′ GAAAAAGCTGA**CAATTG**CTCTTCATTTTATCCT 3′
	3′ CTTTTTCGACT**GTTAAC**GAGAAGTAAAATAGGA 5′
E4	5′ CTCGAGATGTT**CAAATG**GCTTTGAAAGTAA 3′
	3′ GAGCTCTACAA**GTTTAC**CGAAACTTTCATT 5′
E5	5′ GGGTA**CAGATG**CTCGAGATGTT 3′
	3′ CCCAT**GTCTAC**GAGCTCTACAA 5′
M5	5′ GGGTA**TTTTTT**CTCGAGATGTT 3′
	3′ CCCAT**AAAAAA**GAGCTCTACAA 5′

**Figure 10:**
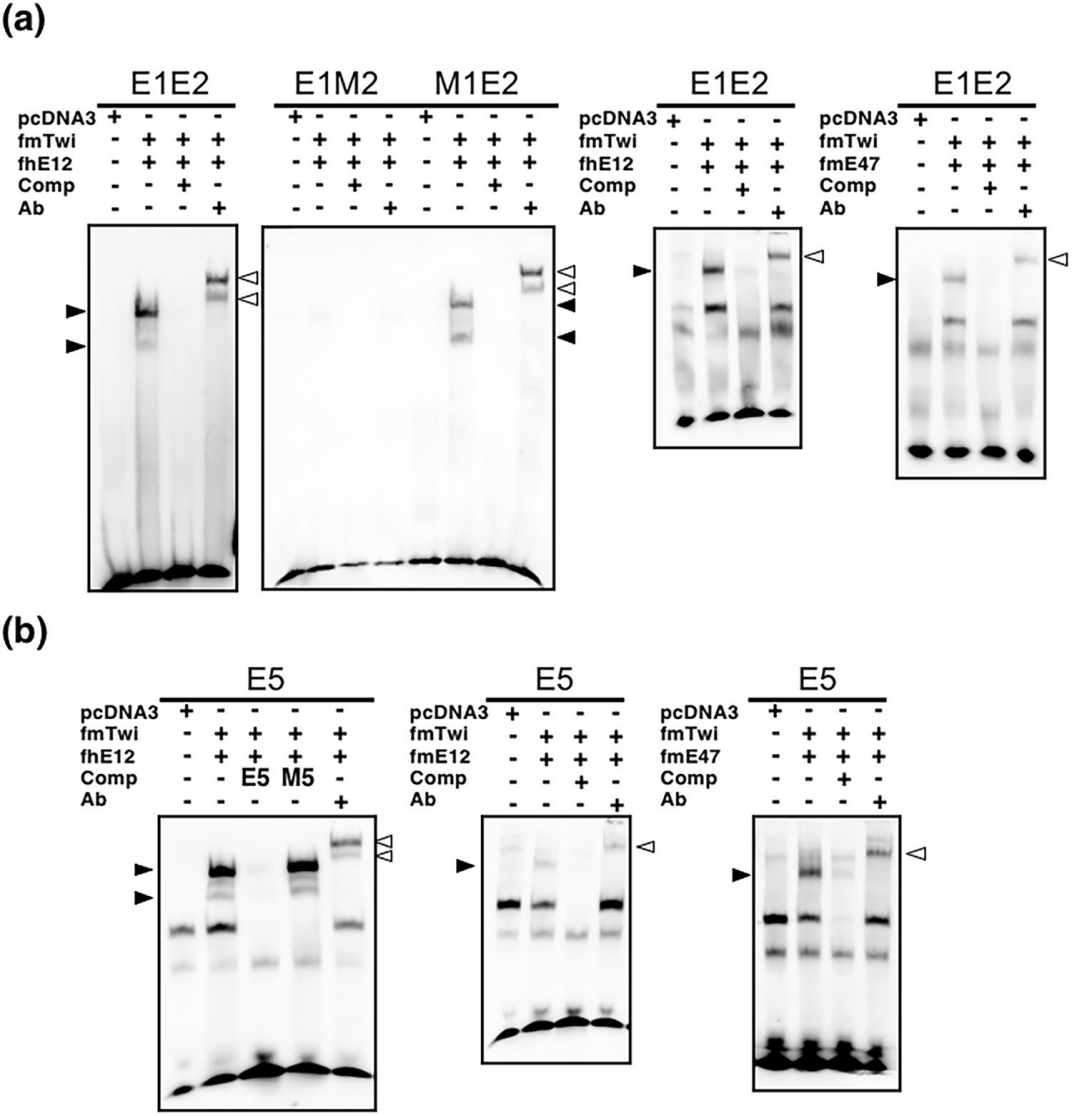
Preferential binding of Twist1/E12 or Twist1/E47 to E2 and E5. Double-stranded synthetic oligonucleotides containing the E-box were end-labelled with biotin and used as a probe for EMSA. The oligonucleotides sequences used in EMSA (E1E2, E1M2, M1E2, and E5) are shown in Table 2. Results shown in (**a**) and (**b**) are from assays using the oligonucleotides containing the consensus E-box sequences (E1E2 and E5) and mutated oligonucleotides (E1M2, M1E2, and M5), respectively. Biotin-labelled probes were incubated with nuclear extracts from HEK293T cells transfected with the empty vector *pcDNA3* as a control, or expression vectors encoding the FLAG fusion of human E12 (fhE12), mouse E12 (fmE12), mouse E47 (fmE47), and mouse Twist1 (fmTwi). Binding reactions were performed in the presence or absence of non-labelled oligonucleotides as binding competitors (Comp) or an anti-FLAG antibody (Ab). The positions of shifted and super-shifted bands are shown with black and hollowed arrowheads, respectively.

We also tested whether Twist1 and/or E12 or E47 bind to E2 (CATCTG) and E5 (CAGATG) by EMSA. EMSA was performed with nuclear extracts containing fmTwi and fhE12, fmE12, or fmE47, using biotin-labelled oligonucleotides containing the E-box and/or the mutated E-box (Table 2). Specific shift bands were detected when fmTwi and fmE12 or fmE47 were incubated with a biotin-labelled E1E2 or E5 oligonucleotide (black arrowheads in Fig. 10a,b). In the presence of an anti-FLAG antibody, supershifted bands were clearly detected (hollowed arrowheads in Fig. 10a,b). Both specific shift bands and supershifted bands were detected when fmTwi and fhE12 were incubated with the biotin-labelled M1E2 oligonucleotide containing a mutated E1 sequence and normal E2 sequence. No specific bands were detected with the biotin-labelled M1M2 or E1M2 oligonucleotides (Fig. 10a). These results suggest that Twist1 and/or E12 or E47 heterodimer complexes directly interact with the oligonucleotide E2 or E5.

Taken together, Scx or Twist1 that dimerizes with E12 or E47 preferentially binds to E2 (CATCTG) and E5 (CAGATG) to transactivate mouse *Tnmd*.

## Discussion

Here, we demonstrated that Scx transactivates the promoter of mouse *Tnmd*, which consists of seven exons encoding a type II transmembrane protein, marking tenocytes and ligamentocytes. Silencing of *Scx* caused marked downregulation of *Tnmd* in cultured tenocytes. Loss of *Scx* function *in vivo* nearly abolished *Tnmd* expression in both tendons and ligaments during musculoskeletal development and growth. Of the five E-boxes around the TATA box, CAGATG and CATCTG are preferential binding sites for Scx, as demonstrated by EMSA. Dual luciferase assays in *Tnmd*-expressing tenocytes revealed enhancer activity upstream of the promoter region. Thus, Scx directly transactivates *Tnmd* via these E-boxes to positively regulate tenocyte differentiation and maturation.

We previously reported that chick *Tnmd* is present at early stages of tendon/ligament formation and is persistently expressed in mature tendons and ligaments at high levels^8^. In mice, our whole-mount *in situ* hybridisation analysis revealed that *Scx* expression in the developing tendon and ligament primordia precedes the expression of *Tnmd*. Later in musculoskeletal development, *Scx* and *Tnmd* are coexpressed with mature tendons and ligaments. Thus, in mice, *Scx* is an early marker gene expressed in both tendon/ligament progenitors and tenocytes/ligamentocytes, whereas *Tnmd* acts as a late marker gene to indicate mature tenocytes/ligamentocytes.

Consistent with our previous report that *Scx* is expressed in a subpopulation of skeletogenic Sox9^+^ progenitor cells that contribute to the formation of future enthesis^18^, *Scx* was also detected in skeletal cells, including chondrogenic ATDC5, osteogenic MC3T3-E1, and chondrocytes, *in vitro*. In contrast, *Tnmd* is not expressed in these skeletogenic cells and is highly specific to mature tenocytes. This is in good accordance with our *in situ* hybridisation data showing that *Tnmd* is detected in the developing tendons and ligaments *in vivo*. Unlike *Col1a2*, *Tnmd* expression was dramatically downregulated during serial passaging of tenocytes, suggesting that *Tnmd* is a good indicator of mature phenotypes in cultured tenocytes. In our rotator cuff injury model using rats, local administration of FGF-2 resulted in biomechanical and histological improvement of the repaired rotator cuff by promoting growth of tenogenic progenitor cells in association with a significant increase in *Tnmd*-positive cells in the midsubstance of the tendon^27^. A strong positive correlation between the location of the aligned collagen fibre orientation and expression levels of *Tnmd* mRNA was observed in this model^27^. In chick embryos, *Tnmd* expression was not detected in oval immature tenocytes, but was observed in elongated mature tenocytes of the embryonic Achilles tendon^8^. Taken together, a high level of *Tnmd* expression is a good indicator of the maturation of tenocytes localized between aligned collagen fibres under both physiological and pathological conditions.

Interestingly, we also detected the expression of *Tnmd* in C3H10T1/2 cells, which is an undifferentiated mesenchymal cell line derived from mouse embryonic fibroblasts. C3H10T1/2 cells have the potential to differentiate into tenocytes^28^. As previously reported, *Tnmd* is also expressed in cultured tendon stem/progenitor cells (TSPC)^19^ and Scx-expressing bone marrow stromal cells^29^. Loss of *Tnmd* results in reduced self-renewal and augmented senescence of tendon/ligament progenitor cells without affecting the multipotential of TSPC^19^. Thus, a low level of *Tnmd* expression may be required for maintenance of the tenogenic potential in stem/progenitor cells *in vitro*.

Scx was originally isolated from a mouse E14.5 cDNA library, using the yeast two-hybrid system as a novel partner of bHLH protein that dimerizes with E12^24^. Additionally, Scx binds to the mouse muscle creatine kinase enhancer (CC**CAGATG**TGGCTGCTCCC) as a heterodimer with E12^24^. It was also reported that Scx acts as a transcriptional activator for *Col1a1* in tendons via binding to the E-box site CACGTG^30^ and *Aggrecan* (*Acan*) in osteosarcoma-derived ROS17/2.8 cells^31^. Scx/E47 heterodimers bind to CAGGTG to regulate *Col2a1* expression together with Sox9 and p300^32^. In this study, we analysed five E-boxes (E1: CACTTG; E2: CATCTG; E3: CAATTG; E4: CAAATG; and E5: CAGATG) upstream and downstream of the TATA box in the mouse *Tnmd* promoter. EMSA revealed that E2 (CATCTG) and E5 (CAGATG) are Scx-binding E-boxes. Li *et al*. performed chromatin immunoprecipitation, to show that Scx binds to the region containing both E4 (CAAATG) and E5 (CAGATG)^28^. In this study, we determined that Scx does not bind to E4 by our EMSA analysis using nuclear extract of HEK293T cells expressing *Scx*. We also found that Twist1 binds to these Scx-binding E-boxes and transactivates the genomic region (−1030 to +84). Thus, not only Scx but also Twist1 regulate *Tnmd* expression via E2 (CATCTG) and E5 (CAGATG) in the promoter region.

Mouse *Scx* gene consists of two exons and is located within the third intron of *block of proliferation 1* (*Bop1*) transcribed in the opposite orientation^33^. The first reported general knockout mice for *Scx* were embryonic lethal, likely because of retention of the *neomycin* phosphotransferase gene linked to phosphoglycerate kinase promoter (*PGK-Neo*) in the targeted allele^34^. Subsequently, Murchison *et al*. generated *Scx* floxed mice and characterised conditional knockout (CKO) mice inactivating *Scx* in the *Prx1* expressing region to demonstrate severe defects in force-transmitting tendon maturation^21^. Similar hypoplastic tendon formation and other phenotypes including defects in ligament and enthesis maturation were also observed in homozygotes with *Scx*^*Cre*^ knock-in allele that we generated by in-frame replacement of most of *Scx* exon 1 with *Cre*^10^. *Scx*^−/−^ mice generated via TALEN-mediated technology in this study were also viable and exhibited similar phenotypes as previously reported *Scx* CKO and *Scx*^*Cre*^ knock-in mice. Mutant mice have only an 11-bp deletion that caused a frameshift, leading to premature stop codons shortly downstream without a further footprint. Thus, this is the first viable general knockout mouse line that is easy to use for generating double or triple knockout mice for tendon and ligament research.

In *Scx*-deficent embryos, severe defects were observed in force-transmitting and intermuscular tendons; however, muscle-anchoring tendons and ligaments were not affected^21^. Homozygous *Scx*^*Cre*^ knock-in mice exhibited defective maturation of tendons and ligaments as well as entheseal and sesamoid cartilage in which *Scx* was transiently expressed during development^10^. Similar phenotypes were observed in *Scx* null mice generated by TALEN-mediated technology. Our *in situ* hybridisation analysis revealed that *Tnmd* expression was nearly absent in both tendons and intracapsular ligaments, such as the anterior cruciate ligaments of *Scx* null mice at P1. Similarly, Tnmd was minimally detected in the distal portion of the Achilles tendon of P1 neonates lacking *Scx*. Silencing of *Scx* in tenocytes isolated from 2-week old rat limb tendons also resulted in significant downregulation of *Tnmd*. These results suggest that *Tnmd* expression in tenocytes is dependent on Scx both *in vivo* and *in vitro*.

Gene and protein expression of Tnmd are nearly absent in *Scx* null embryos, suggesting a crucial role for *Scx* in *Tnmd* expression. However, a loss of *Tnmd* expression was observed in the tenth cultures of tenocytes, although the cells maintained *Scx* expression at a high level. *Scx*^+^ skeletogenic cells, such as MC3T3-E1 and ATDC5 cells, also do not express *Tnmd*. Enhancer activity was increased in the upstream region (−1030 to −295) in tenocytes, but not in NIH3T3 cells not expressing *Tnmd*. As shown in whole-mount *in situ* hybridisation, *Scx* expression precedes that of *Tnmd* during embryogenesis. These results suggest that Scx is necessary for the induction of *Tnmd* expression in mature tenocytes, but not sufficient in immature tenocytes and skeletogenic cells. This is also consistent with our previous finding that retroviral overexpression of *Scx* in the chick hindlimb resulted in significant upregulation of *Tnmd* in tendons but did not induce ectopic *Tnmd* expression outside the tendinous tissue^8^. We speculate that some additional transcription factors are required for the expression of *Tnmd*. Further studies are underway to elucidate other transcriptional factors that coordinately regulate the transcription of *Tnmd* with Scx in tendons and ligaments.

## Methods

### Animals and embryos

Mice and rats were purchased from Japan SLC, Inc. (Hamamatsu, Japan) or CLEA Japan, Inc. (Tokyo, Japan). All animal experimental protocols were approved by the Animal Care Committee of the Institute for Frontier Life and Medical Sciences, Kyoto University, and Committee of Animal Experimentation, Hiroshima University, and conformed to institutional guidelines for the study of vertebrates.

### Generation of TALEN-mediated *Scx*-deficient mice

TALEN plasmids were constructed using the Platinum Gate TALEN Kit (Kit #1000000043, Addgene, Cambridge, MA, USA) as previously described^35^. To prepare TALEN mRNA, TALEN plasmids for *mScx-TALEN-A-L* and *mScx-TALEN-A-R* were linearized with SmaI and purified by phenol-chloroform extraction. *mScx-TALEN-A-L* and *-R* mRNAs were synthesized and a polyA tail was added using the mMESSAGE mMACHINE T7 ULTRA Kit (Ambion, Austin, TX, USA) according to the manufacturer’s instructions. After purification with the MEGAclear kit (Ambion), *mScx-TALEN-A-L* and *mScx-TALEN-A-R* mRNAs were microinjected into the cytoplasm of fertilized eggs obtained from C57BL/6 mice. Injected eggs were transferred into the oviducts of pseudopregnant surrogate ICR female mice. Genomic DNA was extracted from the tail tips of founder mice. A 388-bp fragment of exon 1, which included recognition sites for TALENs, was amplified by PCR using primers (*Scx_GTF3*: 5′-GCCTGTGGGGACCTAAAGAG-3′; *Scx_GTR4*: 5′-TCGGTGGGGATGAGTGTGCGCAGCGC-3′). The amplified fragment was then used for direct sequencing. Sequencing was performed with the BigDye Terminator Cycle Sequencing kit and an ABI 3100 Genetic Analyzer (Applied Biosystems, Foster City, CA, USA).

### Cell culture

Mouse limb and tail tendons were isolated from 4-week-old male mice and seeded onto 100-mm cell culture dishes. Tenocytes outgrown from the tendon were passaged once or twice and grown in minimum essential medium Eagle alpha modification (α-MEM) supplemented with 10% foetal bovine serum (FBS). Rat tenocytes were isolated from limb tendons of 7- or 14-day-old male Wistar rats. Minced tendons were incubated with 0.1% ethylenediaminetetraacetic acid (EDTA) (Dojin, Tokyo, Japan) at 37 °C for 20 min and digested with 0.05% trypsin containing 0.53 mM EDTA (Gibco, Grand Island, NY, USA) at 37 °C for 5 min followed by digestion with 0.1% collagenase (Roche, Basel, Switzerland) at 37 °C for 10 min. Tenocytes were grown in α-MEM supplemented with 10% FBS (Cambrex, East Rutherford, NJ, USA) and 50 μg/mL kanamycin (Sigma-Aldrich, St. Louis, MO, USA) on dishes coated with type I collagen (Koken, Tokyo, Japan). Rat chondrocytes were isolated from rib cartilages of 4-week-old Wistar rats. Cartilage minces were incubated with 0.1% EDTA at 37 °C for 20 min and digested with 0.15% trypsin (Difco, Detroit, MI, USA) at 37 °C for 1 h and 0.1% collagenase at 37 °C for 3 h. Chondrocytes were grown in a 1:1 mixture of Dulbecco’s modified Eagle’s medium and Ham’s F-12 medium (DMEM/F12 medium; Asahi Techno Glass, Haibara, Japan) supplemented with 10% FBS (Sigma-Aldrich). NIH3T3 cells were grown in DMEM (Sigma-Aldrich) supplemented with 10% FBS (Biological Industries, Beit Haemek, Israel) and 50 μg/mL kanamycin. C3H10T1/2 cells were grown in DMEM (Sigma-Aldrich) supplemented with 10% FBS (Sigma-Aldrich). MC3T3-E1 cells were grown in α-MEM supplemented with 10% FBS. ATDC5 cells were grown in DMEM/F12 medium (Asahi Techno Glass) supplemented with 5% FBS (Hana-Nesco Bio Corp., Tokyo, Japan), 10 μg/mL human insulin (Roche), 10 μg/mL human transferrin (Roche), and 3 × 10^−8^ M sodium selenite (Sigma-Aldrich), as described previously^36^. Cells were incubated at 37 °C in a 5% CO_2_ atmosphere.

### *Scx* knockdown by RNA interference

Small interfering RNA (siRNA) oligonucleotide duplexes were purchased from GE Healthcare Life Sciences (Little Chalfont, UK). *Scx* was depleted using *siScx-1* (J-113656-09) and *siScx-2* (J-113656-10) included in the ON-TARGET plus rat Scx siRNA set (GE Healthcare Life Sciences, LQ-113656-00-002). For the control experiment, siGENOME non-targeting siRNA pool no. 1 (D-001206-13-05) was used. Transfection of siRNA into rat tenocytes was performed with DharmaFECT 1 transfection reagent (GE Healthcare Life Sciences) according to the manufacturer’s instructions.

### Quantitative RT-PCR (qRT-PCR) analysis

Total RNA was extracted from rat tenocytes, using an RNeasy Plus Mini kit (Qiagen, Hilden, Germany). Two-hundred nanograms of total RNA was used to synthesize cDNA with a PrimeScript RT reagent kit (Takara Bio, Shiga, Japan). qRT-PCR was performed using SYBR Premix Ex Taq II (Takara Bio) on a StepOne instrument (Life Technologies, Carlsbad, CA, USA). Relative mRNA expression was normalized to that of *18 S rRNA* and calculated using the 2^−ΔΔCT^ method. Specific primers for qRT-PCR are listed in Table 3.

**Table 3:** Primers for qRT-PCR.

		Sequence (5′–3′)
*Col1a2*	Forward	ACTCAGCCACCCAGAGTGGAA
	Reverse	TTGACAGGTTGGGCCTGGA
*Scx*	Forward	AGCCCAAACAGATCTGCACCTT
	Reverse	CTTCCACCTTCACTAGTGGCATCA
*Tnmd*	Forward	ATGGGTGGTCCCACAAGTGAA
	Reverse	CTCTCATCCAGCATGGGATCAA
*18* *S rRNA*	Forward	AAGTTTCAGCACATCCTGCGAGTA
	Reverse	TTGGTGAGGTCAATGTCTGCTTTC

### Northern blot analysis

Total RNA was prepared from cultured tenocytes, chondrocytes, NIH3T3 cells, C3H10T1/2 cells, ATDC5 cells, and MC3T3-E1 cells as described previously^25^. Total RNA (15 μg) was denatured with 6% formaldehyde, electrophoresed on a 1% agarose gel, and transferred onto Nytran membranes with Turboblotter (Schleicher and Schuell, Whatman plc, Maidstone, UK). Rat *Tnmd* and rat *Scx* probes were amplified from cDNA prepared from cultured rat tenocytes with SuperScript II reverse transcriptase (Invitrogen, Carlsbad, CA, USA). Probes for mouse *Tnmd*, mouse *Scx*, and rat type II collagen alpha 1 (*Col2a1*) were described previously^10,25,36^. A probe for rat type I collagen alpha 2 (*Col1a2*) was generously provided by Dr. Bjorn Olsen. A probe for mouse *Twist1* was amplified from a cDNA clone purchased from Open Biosystems (Lafayette, CO, USA). Hybridisation was performed overnight at 42 °C with an appropriate cDNA probe labelled with [α-^32^P]dCTP in a solution containing 50% formamide (Wako, Osaka, Japan), 6 X saline-sodium phosphate-EDTA buffer (Sigma-Aldrich), 0.1% bovine serum albumin (Sigma-Aldrich), 0.1% Ficoll 400 (GE-Healthcare), 0.1% polyvinylpyrrolidone (Wako), 0.5% sodium dodecyl sulphate (Wako), and 100 μg/mL denatured salmon sperm DNA (Wako).

### *In situ* hybridisation

For whole-mount *in situ* hybridisation, mouse embryos were fixed in 4% paraformaldehyde/phosphate-buffered saline (PBS) overnight and dehydrated with methanol. After rehydration, the embryos were treated with proteinase K (Invitrogen) and hybridised with DIG-labelled probes at 70 °C overnight. For *in situ* hybridisation analysis of frozen sections, embryos and neonates were infiltrated with 18% sucrose/PBS, frozen, and cryosectioned at a thickness of 8 μm. The sections were post-fixed with 4% paraformaldehyde /PBS, incubated in 0.1% diethylpyrocarbonate/PBS, and hybridised with DIG-labelled probes at 50 °C overnight. After washing, the hybridised RNAs were detected with alkaline phosphatase conjugated anti-DIG antibody (Roche) and BM purple (Roche). The antisense or sense RNA probes for mouse *Tnmd*^9^, mouse *Scx*^37^, and mouse *Col1a1*^25,38^ were transcribed from their respective plasmids with a digoxygenin (DIG) RNA labelling kit (Roche).

### Immunostaining

Mouse neonates were infiltrated with 18% sucrose/PBS, frozen, and cryosectioned at a thickness of 8 μm. After post-fixation with ice-cold acetone for 5 min, the sections were incubated with 3.2% skim milk/PBS for 20 min and incubated overnight at 4 °C with primary antibodies diluted with 3.2% skim milk/PBS. The sections were then incubated with appropriate secondary antibodies conjugated with Alexa Fluor 488 or 594 (Life Technologies). The primary antibodies used were anti-Tnmd (diluted 1:1000)^6^, anti-type I collagen (Col1) (Rockland Immunochemicals, Limerick, PA, USA; 600-401-103; 1:500), and anti-Chmd (Cosmo Bio, Tokyo, Japan; TCS-005; 1:1000), respectively. Nuclei were counterstained with 4′,6-diamidino-2-phenylindole. Images were captured under a Leica DMRXA microscope equipped with a Leica DC500 camera (Leica Microsystems, Wetzlar, Germany).

### Determination of transcriptional start sites

The transcriptional start sites of the mouse *Tnmd* gene were determined by the CapSite hunting method with 16-day-old mouse embryo CapSite cDNA (Nippon Gene, Toyama, Japan), according to the manufacturer’s instructions. CapSite cDNAs were used as a template for the first round of polymerase chain reaction (PCR) with a forward primer (1RC: 5′-CAAGGTACGCCACAGCGTATG-3′) and mouse *Tnmd* gene-specific reverse primer (mTnmd-R1; 5′-CACTGGTAGGAAAGTGAAGATCTTC-3′). Samples were amplified for 35 cycles under the following conditions: denaturation for 30 s at 94 °C, annealing for 30 s at 55 °C, and extension for 30 s at 72 °C. Products of the first round of PCR were used as the template in the second round of nested PCR. Nested PCR was performed with a forward primer (*2RC*: 5′-GTACGCCACAGCGTATGATGC-3′) paired with the mouse *Tnmd* gene-specific reverse primers (*mTnmd-R2*: 5′-CACCGTCCTCCTCAAAGTCCTG-3′; *mTnmd-R3*: 5′-CATTCTCATCTATTTCTTCCTCTGG-3′; and *mTnmd-R4*: 5′-CACTGACTGTTCAAAGAAAGTTG-3′). Samples were amplified for 35 cycles under the following conditions: denaturation for 30 s at 94 °C, annealing for 30 s at 57 °C, and extension for 30 s at 72 °C. The amplified products were cloned into *pCRII-TOPO* (Invitrogen) and sequenced with the 310 Genetic Analyzer (Applied Biosystems).

### Construction of expression vectors

The entire coding sequence of *mScx* was amplified from mScx cDNA (Cserjesi *et al*.^24^) using LA Taq polymerase (Takara) with a forward primer (*mScxF*) and reverse primer (*mScxR*) for *mScx* and subcloned into *pCRII-TOPO* (*mScxpCRII*; Invitrogen). FLAG-tagged *mScx* was then amplified from *mScxpCRII* with the forward primer containing the coding sequence of mScx followed by the FLAG peptide coding sequences with a NotI site (*FLAGmScxF*) and a specific reverse primer (*FLAGmScxR*) using PrimeStar DNA polymerase (Takara). FLAG-tagged *mScx* was inserted into the EcoRV site of *pcDNA3* vector (Invitrogen) to construct *pcDNA3-FLAG-mScx*. For construction of FLAG-tagged mouse E12 (fmE12) and mouse E47 (fmE47), a DNA fragment from the second codon to the stop codon were amplified with a forward primer containing a NotI site (*FLAGmE12*/*47 F*) and reverse primer with a NotI site (*FLAGmE12*/*47 R*). To construct FLAG-tagged mTwist1 (fmTwi), a DNA fragment from the second codon to the stop codon was amplified with a forward primer containing a NotI site (*FLAGmTwist1F*) and reverse primer with a NotI site (*FLAGmTwist1R*). The amplified fragments were inserted in place of the *mScx* fragment of *pcDNA3-FLAG-mScx*. The mouse *E12* (BC018260), mouse *E47* (BC006860), and mouse *Twist1* (BC083139) cDNA clones for PCR amplification were purchased from Open Biosystems. Primers used for construction of expression vectors are shown in Supplementary Table 1.

### Transient transfection and dual luciferase assay

Rat tenocytes were transiently transfected with *pGL4*.*10[luc2]* (Promega, Madison, WI, USA) or *pGL4*.*23[luc2*/*minP]* (Promega) reporters using Lipofectamine LTX (Invitrogen). Each reporter construct was co-transfected with *pGL4*.*74[hRluc*/*TK]*. For co-transfection experiments, reporter and expression vectors were transfected in a 96-well plate format. After 24 h, luciferase activities were measured using PicaGene Dual Sea Pansy Luminescence kit (TOYO INC MFG CO., LTD., Tokyo, Japan) and microplate luminometer (Berthold Technologies, Bad Wildbad, Germany; Centro XS^3^ LB960). Reporter construct activity was normalized by comparison with the activity of the Renilla luciferase construct. All experiments were performed in triplicate.

### Electrophoretic mobility shift assay (EMSA)

The sequences of the oligonucleotides containing the E-box and/or mutated E-box are shown in Table 2. Double-stranded oligonucleotide probes were labelled with biotin using the Biotin 3′End DNA Labeling kit (Thermo Scientific, Waltham, MA, USA). Nuclear proteins were isolated using a Nuclear Extract kit (Active Motif, Carlsbad, CA, USA) from HEK293T cells or Lenti-X HEK293T Cells (Takara Bio) transfected with *pcDNA3* or *pcDNA3*.*1* vectors expressing fmScx, fmTwi, fhE12, fmE12, or fmE47 proteins following the manufacturer’s protocol. *pcDNA3*.*1* vector with fhE12 was kindly provided by Dr. Eric N. Oloson^39^. DNA-protein binding was assayed with a Gelshift Chemiluminescent EMSA kit (Active Motif) following the manufacturer’s protocol. Briefly, a total of 2 μL of nuclear proteins were incubated with 100 fmol of biotin-labelled DNAs for 30 to 90 min at room temperature in binding buffer containing 1 μg of poly d(I-C), 5 mM MgCl_2_, and 2.5% glycerol. For competition experiments, a 200-fold or 400-fold molar excess of unlabelled oligonucleotide was included in the binding reaction. Antibody supershifts were carried out by adding 5 μg of anti-FLAG M2 monoclonal antibody (Sigma-Aldrich) together with nuclear extracts prior to incubation with DNA. Protein-DNA complexes were resolved by electrophoresis on 5% or 4% polyacrylamide gel in Tris-borate-EDTA buffer, transferred onto Nytran SPC membrane (GE Healthcare), and detected with streptavidin-horseradish peroxidase conjugate and chemiluminescent reagent.

### Statistical analysis

*P*-values were calculated by one-way analysis of variance using the SPSS software package (SPSS 21.0, SPSS, Inc., Chicago, IL, USA). The data were considered statistically significant at a *P* value < 0.05.

## Electronic supplementary material

Supplementary information
