# Supplementary material for: Scleraxis is a transcriptional activator that regulates the expression of Tenomodulin, a marker of mature tenocytes and ligamentocytes
Source: Sci Rep. 2018 Feb 16;8:3155. doi: 10.1038/s41598-018-21194-3 (PMC5816641; doi:10.1038/s41598-018-21194-3)
Supplement: Supplementary file 1 — Supplementary information [file 41598_2018_21194_MOESM1_ESM.pdf]

Scleraxis is a transcriptional activator that regulates the expression of Tenomodulin, a marker of mature tenocytes and ligamentocytes

Chisa Shukunami[1]*, Aki Takimoto[2], Yuriko Nishizaki[2§], Yuki Yoshimoto[1], Seima Tanaka[1], Shigenori Miura[2], Hitomi Watanabe[3], Tetsushi Sakuma[4], Takashi Yamamoto[4], Gen Kondoh[3], and Yuji Hiraki[2]

[1]Department of Molecular Biology and Biochemistry, Division of Dental Sciences, Graduate School of Biomedical & Health Sciences, Hiroshima University, Hiroshima 734-8553, Japan

[2]Laboratory of Cellular Differentiation, Institute for Frontier Life and Medical Sciences, Kyoto University, Kyoto 606-8507, Japan

[3]Laboratory of Integrative Biological Science, Institute for Frontier Life and Medical Sciences, Kyoto University, Kyoto 606-8507, Japan

[4]Department of Mathematical and Life Sciences, Graduate School of Science, Hiroshima University, Higashi-Hiroshima, Hiroshima, Japan

[§]Current address: Functional Morphology Laboratory, Department of Clinical Pharmacy, Faculty of Pharmacy, Yokohama University of Pharmacy, Yokohama, Japan

*Address all correspondence to Chisa Shukunami, DDS, Ph.D.

Department of Molecular Biology and Biochemistry, Division of Dental Sciences,

Graduate School of Biomedical & Health Sciences, Hiroshima University,

Hiroshima 734-8553, Japan

Tel: +81-82-257-5628

Fax: +81-82-257-5629

E-mail: shukunam@hiroshima-u.ac.jp

Supplementary Table 1. PCR primers used for construction of expression vectors

Name	Sequence
FLAGmScxF	5'-ACCATGGACTACAAAGACGATGACGACAAGGCGGCCGCGTCCTTCGCCATGCTGCGTTCAGCG-3'
FLAGmScxR	5'-CCCTAACTTCGAATCGCCGTCTTTC-3'
FLAGmE12/47F	5'-ATTTGCGGCCGCGAACCAGTCTCAGAGAATGGCAC-3'
FLAGmE12/47R	5'-ATTTGCGGCCGCTCACAGGTGCCCGGCTGGGTTG-3'
FLAGmTwist1F	5'-ATTTGCGGCCGCGCAGGACGTGTCCAGCTCGCCAGTCTC-3'
FLAGmTwist1R	5'-ATTTGCGGCCGCCTAGTGGGACGCGGACATGGACCAGGCC-3'

Supplementary Figure 1

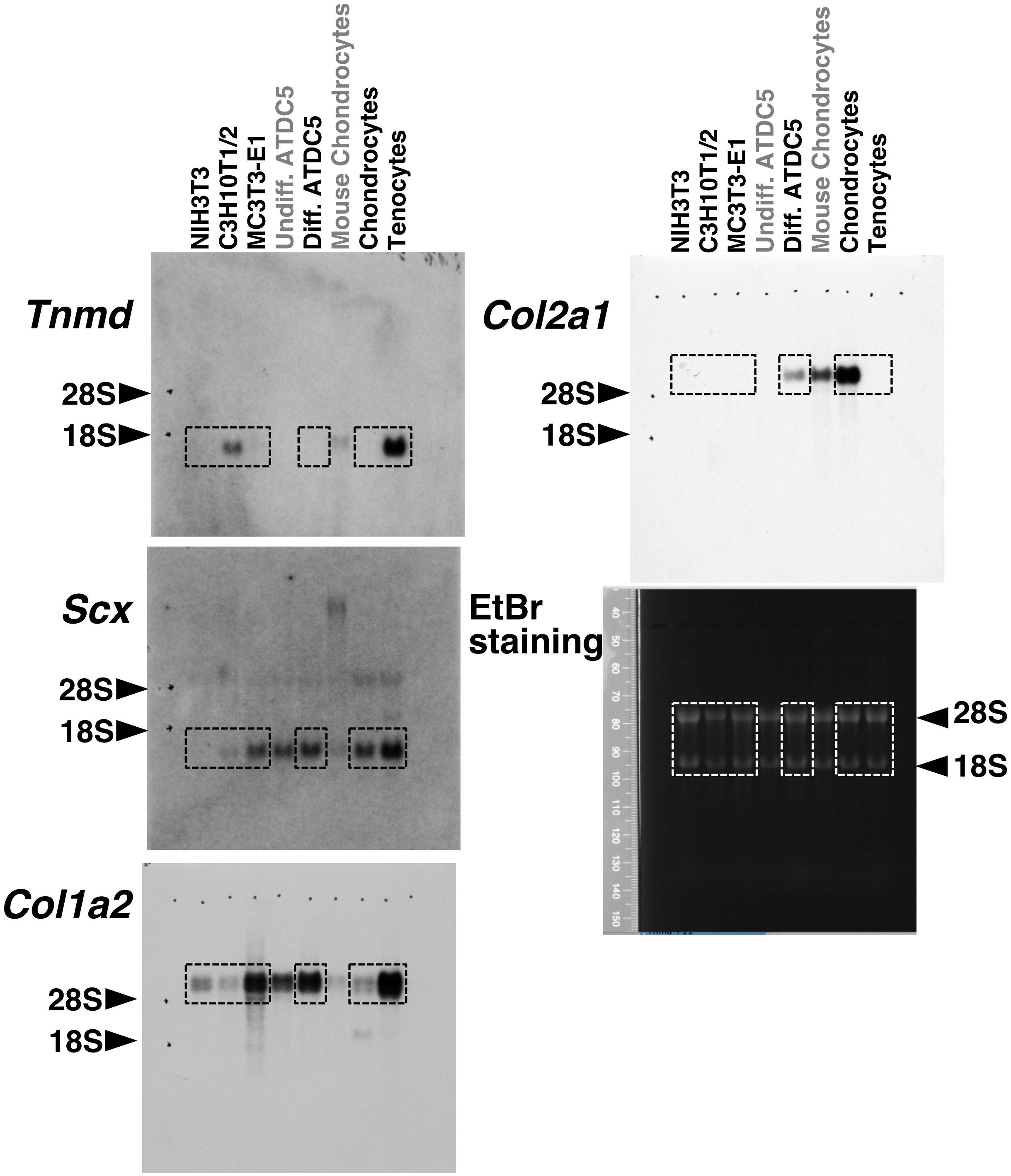

Supplementary Figure 1.
Scanned images of northern blots developed on X-ray films and RNA gel electrophoresis in the main manuscript (Figure1g) are shown. The cropped images are enclosed with dotted lines. Arrowheads indicate the positions of the ribosomal RNA subunits.

Supplementary Figure 2

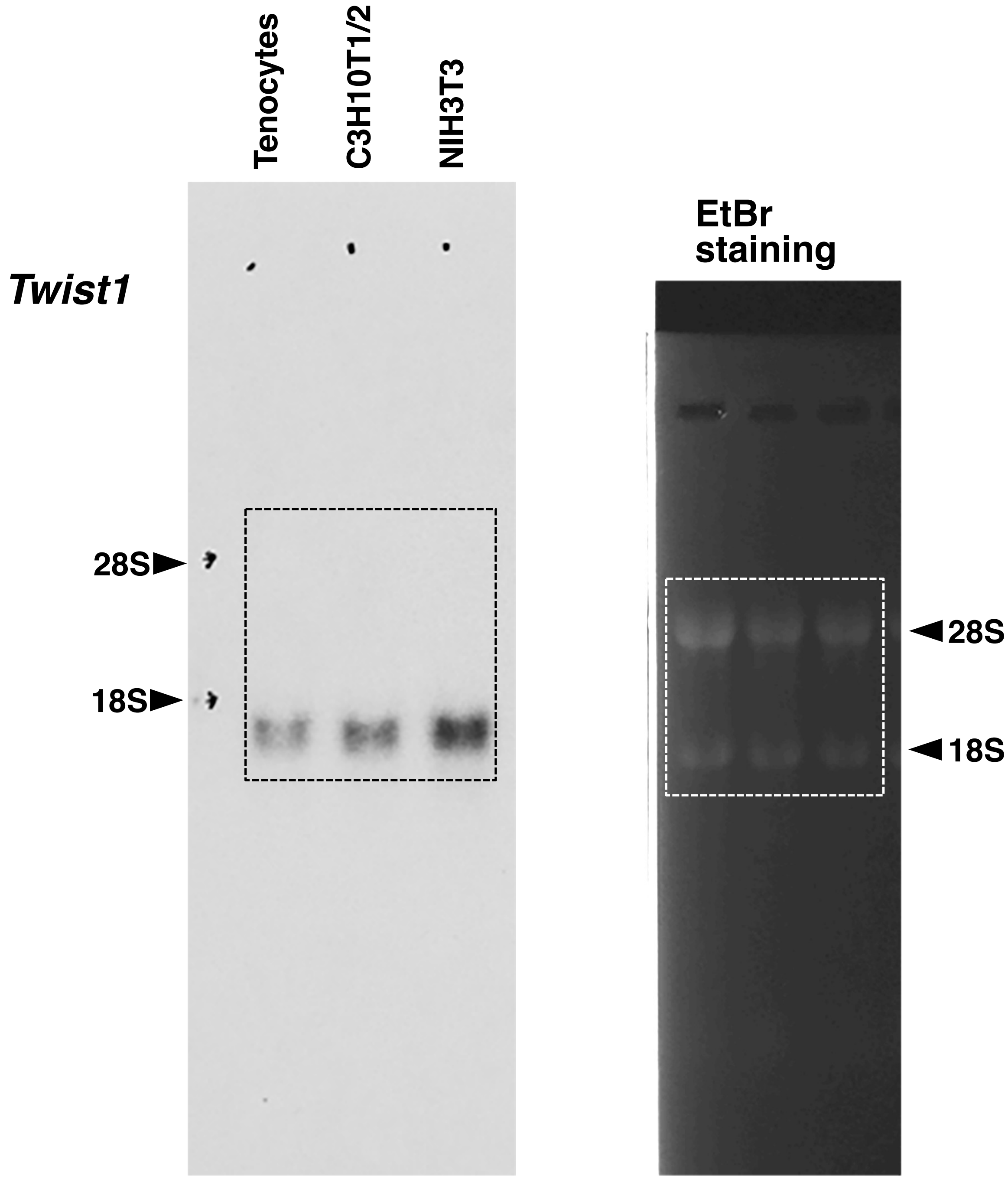

Supplementary Figure 2.
A scanned image of a northern blot developed on X-ray films and RNA gel electrophoresis in the main manuscript (Figure1h) are shown. The cropped images are enclosed with dotted lines. Arrowheads indicate the positions of the ribosomal RNA subunits.

Supplementary Figure 3

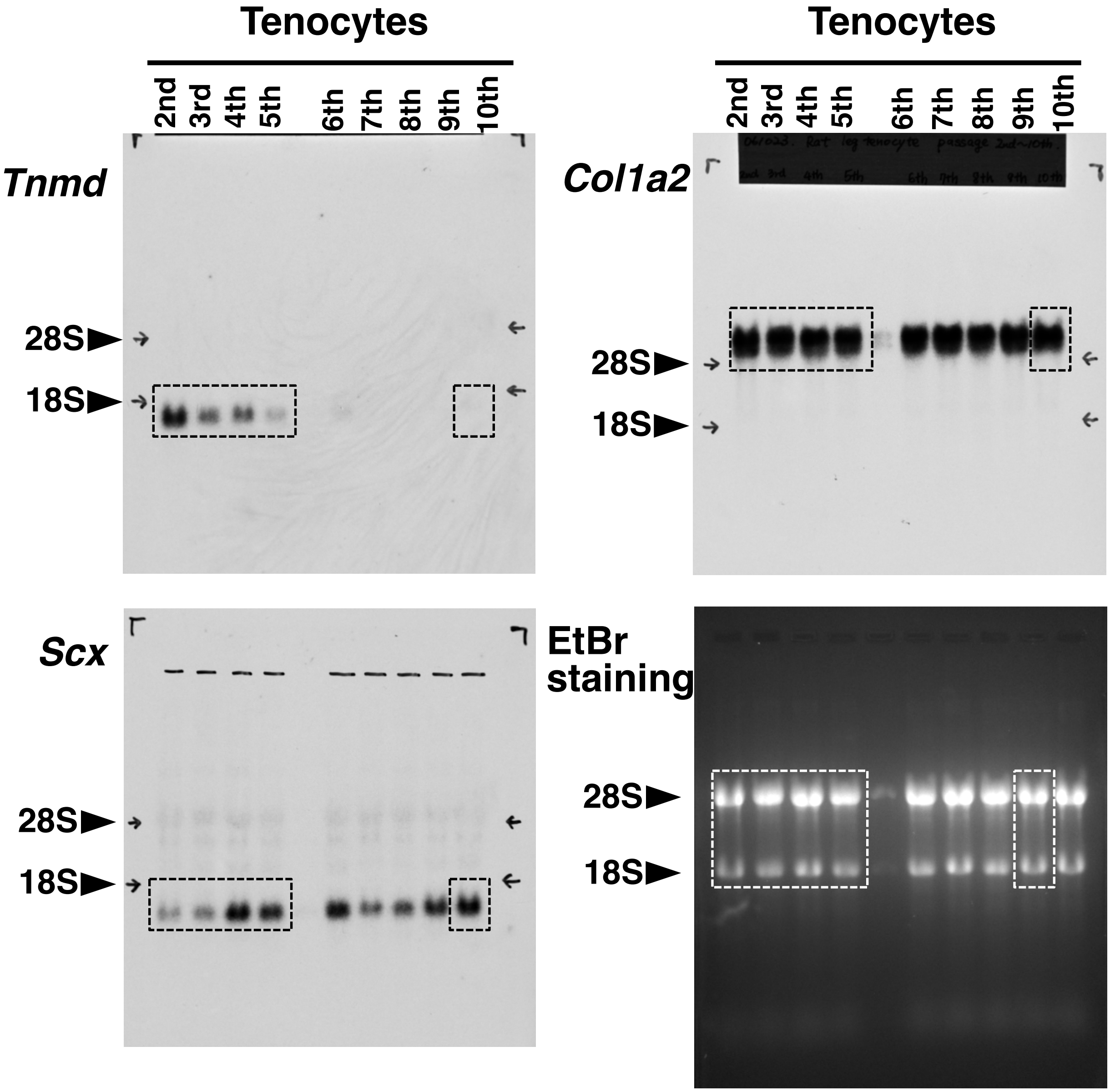

Supplementary Figure 3.
Scanned images of northern blots developed on X-ray films and RNA gel electrophoresis in the main manuscript (Figure1i) are shown. The cropped images are enclosed with dotted lines. Arrowheads indicate the positions of the ribosomal RNA subunits.
